# Regulation of Transcriptional Networks by PKC Isozymes: Identification of c-Rel as a Key Transcription Factor for PKC-Regulated Genes

**DOI:** 10.1371/journal.pone.0067319

**Published:** 2013-06-27

**Authors:** Rachana Garg, M. Cecilia Caino, Marcelo G. Kazanietz

**Affiliations:** Department of Pharmacology, Perelman School of Medicine, University of Pennsylvania, Philadelphia, Pennsylvania; Northwestern University, United States of America

## Abstract

**Background:**

Activation of protein kinase C (PKC), a family of serine-threonine kinases widely implicated in cancer progression, has major impact on gene expression. In a recent genome-wide analysis of prostate cancer cells we identified distinctive gene expression profiles controlled by individual PKC isozymes and highlighted a prominent role for PKCδ in transcriptional activation.

**Principal Findings:**

Here we carried out a thorough bioinformatics analysis to dissect transcriptional networks controlled by PKCα, PKCδ, and PKCε, the main diacylglycerol/phorbol ester PKCs expressed in prostate cancer cells. Despite the remarkable differences in the patterns of transcriptional responsive elements (REs) regulated by each PKC, we found that c-Rel represents the most frequent RE in promoters regulated by all three PKCs. In addition, promoters of PKCδ-regulated genes were particularly enriched with REs for CREB, NF-E2, RREB, SRF, Oct-1, Evi-1, and NF-κB. Most notably, by using transcription factor-specific RNAi we were able to identify subsets of PKCδ-regulated genes modulated by c-Rel and CREB. Furthermore, PKCδ-regulated genes condensed under the c-Rel transcriptional regulation display significant functional interconnections with biological processes such as angiogenesis, inflammatory response, and cell motility.

**Conclusion/Significance:**

Our study identified candidate transcription factors in the promoters of PKC regulated genes, in particular c-Rel was found as a key transcription factor in the control of PKCδ-regulated genes. The deconvolution of PKC-regulated transcriptional networks and their nodes may greatly help in the identification of PKC effectors and have significant therapeutics implications.

## Introduction

It has been widely acknowledged that protein kinase C (PKC) plays important roles in the development and progression of cancer. The PKC family comprises at least 10 related serine-threonine kinases with extensive functional diversity, and it has been classified into classical (cPKCs α, βI, βII and γ), novel (nPKCs δ, ε, η and θ), and atypical (aPKCsζ and λ/ι) based on the structural and biochemical properties of the different isozymes [1], [2]. As extensively reported over the last two decades, PKCs are important constituents of signaling pathways that control mitogenesis, differentiation, survival, adhesion, motility, and metastatic dissemination of cancer cells. It is also well accepted that individual PKC isozymes differentially control these cellular functions, in some cases having overlapping roles and in others completely opposite functions. Although the mechanisms behind this functional diversity are only partially understood, it is fully recognized that PKC isozymes signal via different signaling cascades as a consequence of their differential access to intracellular compartments and substrates upon activation [1], [3], [4], [5].

Studies in multiple cellular models established that activation of PKC isozymes has major impact on the expression of genes and gene products. Since the 1980’s it is known that phorbol esters, natural compounds that activate cPKCs and nPKCs by mimicking the action of the lipid second messenger diacylglycerol (DAG), strongly influence the transcriptional activation of genes. Early studies from the Karin laboratory amongst others defined *cis*-acting promoter elements known as “TPA-responsive elements” or TREs, which were originally defined as binding sites for the AP-1 transcription factor [6], [7]. The fact that PKC isozymes act as effectors of multiple tyrosine-kinases, GPCRs, cytokine receptors, and adhesion receptors, together with their ability to regulate a vast number of signaling pathways, including the MEK/Erk, JNK, p38, NF-κB, and JAK/Stat cascades, strongly argues for a complex relationship with transcriptional responses. Indeed, PKCs modulate the expression and/or activity of several transcription factors, including c-Fos, Jun, NF-κB, Stats, and p53 [8], [9], [10], [11], [12], [13], [14]. Based on the large diversity of PKC effectors, one would predict an exceptionally intricate paradigm where each PKC controls the expression of different subsets of genes. Unfortunately, at the present time there is very little information on how individual members of the PKC family regulate gene expression. Moreover, the relative contribution of transcriptional responses to the overall cellular effects triggered by activation of PKC isozymes remains ill-defined.

In a recent study, our laboratory reported the first PKC isozyme-specific analysis of global gene expression [15]. Using as a model LNCaP prostate cancer cells subject to specific RNAi depletion for PKCα, PKCδ, and PKCε (the main DAG/phorbol ester-responsive PKCs expressed in these cells), this microarray analysis revealed that PKC isozymes exhibit both overlapping and selective roles in the control of gene expression, as one would anticipate from their distinctive functional properties. In particular, a prominent role for PKCδ was established as a mediator of gene induction by phorbol esters relative to the other PKCs. Moreover, we identified a subset of genes that contribute to PKCδ-mediated apoptotic responses by phorbol esters and chemotherapeutic agents, strongly arguing for the involvement of a transcriptional component in responses mediated by this nPKC. The diversity in the gene expression response by PKC isozymes presumably signifies the complexity of the molecular routes and regulatory networks regulated by each member of the family. Hence, delineation of the transcriptional networks governing PKC-mediated gene regulation would provide new insight into the mechanisms of PKC-mediated biological responses and in addition uncover converging network nodes that potentially control signaling pathways associated with human cancer progression.

Based on our previous microarray data of PKCα-, PKCδ-, and PKCε-regulated genes [15], in the present study we carried out a thorough bioinformatics analysis aimed at characterizing the transcriptional networks involved in gene expression mediated by individual PKC isozymes in prostate cancer cells. Candidate transcription factors in the promoters of genes regulated by each PKC were identified, some of them known to have prominent roles in the progression of prostate cancer and other cancers. In particular, our analysis established c-Rel as a key transcription factor in the control of PKCδ-regulated genes.

## Results

### Transcriptional Network Analysis of PKCα-, PKCδ- and PKCε-regulated Genes Reveals Gene Set-specific Clusters of Transcription Response Elements (REs)

**Figure 1:**
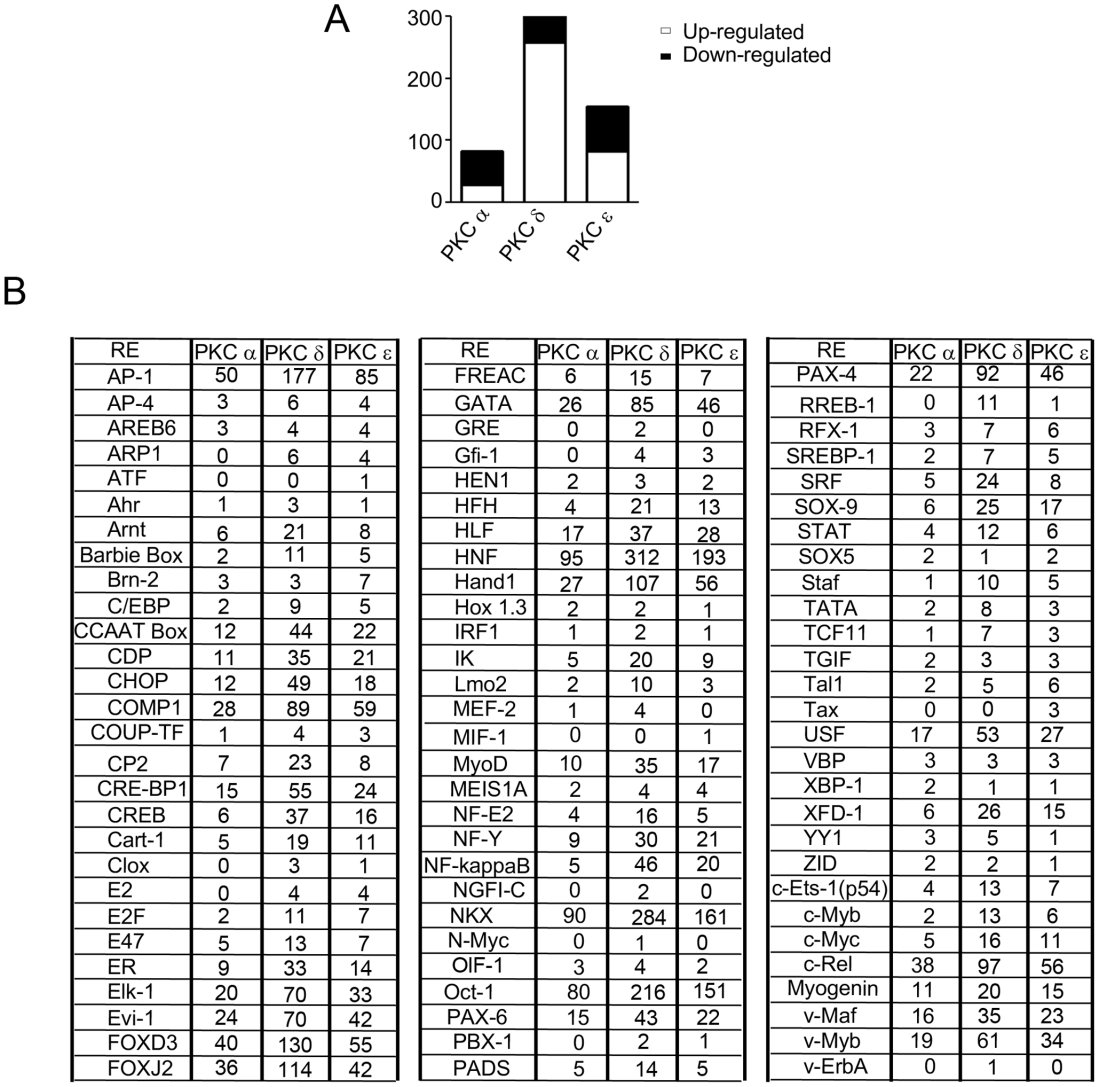
Relative contribution of PKC isozymes to the gene expression by PMA. (A) Total number of PMA-regulated genes that are either up-regulated or down-regulated by each PKC isozyme as revealed by our earlier microarray analysis [15]. (B) Frequency of occurrence of each transcriptional response element (*RE*) in the promoter region of PKC isozymes was determined using the Promoter Analysis and Interaction Network Toolset (PAINT).

In our previous study we carried out a comprehensive analysis of genes controlled by DAG/phorbol ester-regulated PKC isozymes by microarray using a prostate cancer cell line as a model [15]. This analysis, in which individual PKCs were silenced from LNCaP prostate cancer cells using RNAi, revealed a characteristic pattern of gene expression controlled by PKCα, PKCδ and PKCε. Results from that study also established PKCδ as the most relevant isoform in controlling the induction of genes by phorbol esters, including a number of genes implicated in apoptosis that were induced by PKCδ activation. As seen in **Figure 1A** (see also [15]), PKCδ predominantly controls the induction of genes by PMA relative to PKCα or PKCε.

In order to identify transcription factor binding sites in the promoter regions of PKCα-, PKCδ-, or PKCε-regulated genes we used the Promoter Analysis and Interaction Network Tool (PAINT v4.0). Gene sets for each PKC isozyme representing those genes whose expression is either up- or down-regulated by PMA by 2-fold were assembled and subsequently filtered if their expression is either abridged or enhanced by ≥ 50% as a result of isozyme-specific PKC depletion. A comprehensive list of genes regulated by PKCα, PKCδ, or PKCε is presented in **Tables S1, S2, and S3**, respectively. The Entrez Gene ID (Locuslink) of each of these genes was separately provided as the inputs to PAINT to retrieve their putative promoter sequences (5000 bp) and analyze them for the presence of transcription factor response elements (RE). For PKCα, PKCδ- and PKCε-regulated genes, the Upstreamer module of PAINT fetched a list of 107, 338 and 179 unique promoters, respectively. These promoter sequences were further processed using the TREretriever module combined with the publicly available program Match-TRANSFAC (www.gene-regulation.com), which retrieved motif matches for 112, 141 and 135 distinct REs for PKCα, PKCδ and PKCε, respectively. Using the Feasnet Viewer module of PAINT we then converted these data into graphic representations of the RE network (Candidate Interaction Matrix, CIM) for PKCα, PKCδ and PKCε as illustrated in **Figures S1, S2, and S3**, respectively. This analysis identified those REs that are statistically over- or under-represented on the promoters of PKCα, PKCδ or PKCε-regulated genes, as compared to their occurrence with the larger background set of promoters (Human Genome U133A_2.0 Array). Analysis of occurrence of REs as obtained from the CIM analysis revealed that their frequencies were higher for PKCδ-regulated genes than for PKCε- or PKCα-regulated genes **(** **Figure 1B** **)**.

### Enrichment Analysis of Over-represented REs in Genes Regulated by PKC Isozymes

**Figure 2:**
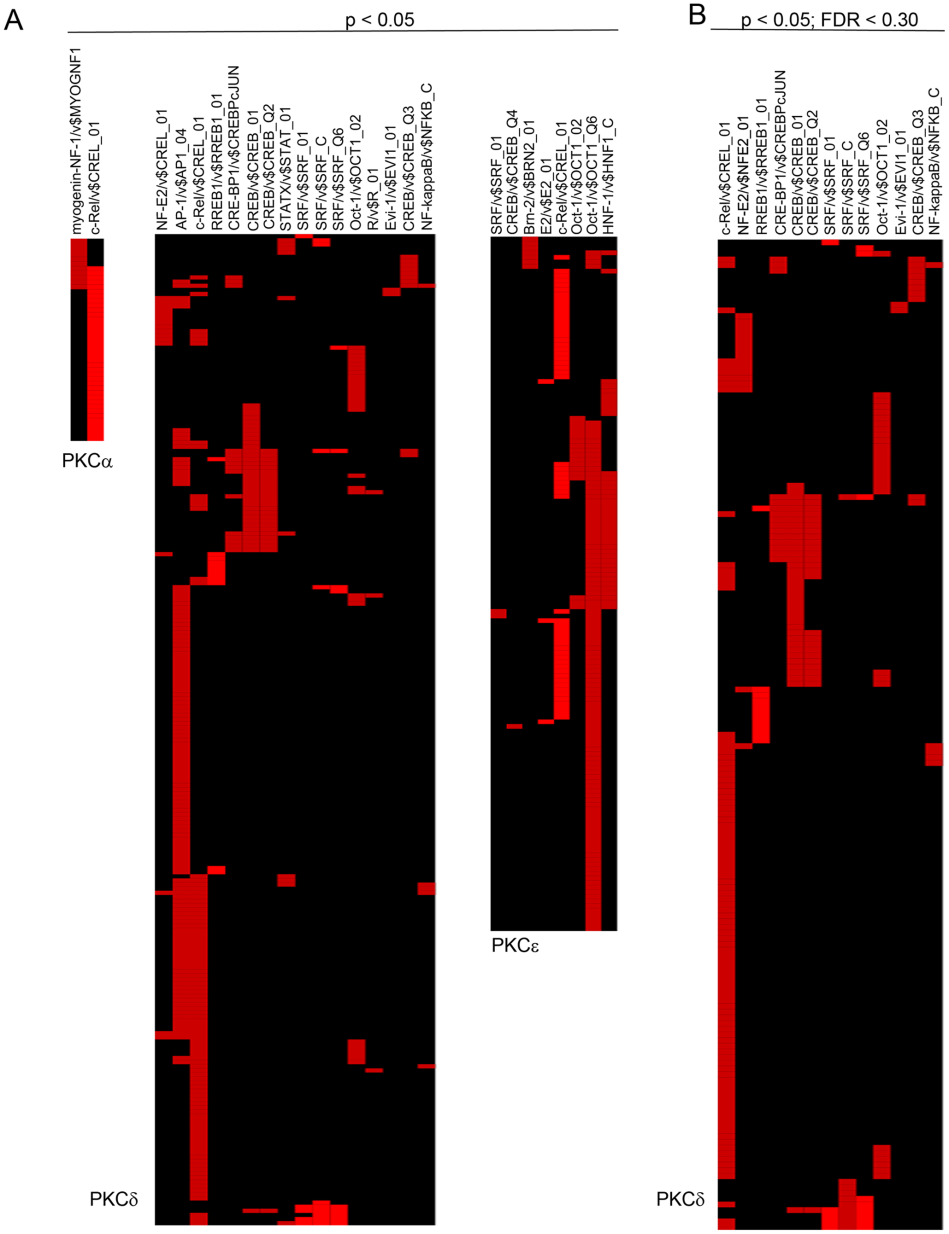
Candidate Interaction Matrix for statistically enriched REs for each PKC isozyme, as determined by PAINT. *Columns* correspond to the TRANSFAC identifiers for each over-represented RE. *Rows* represent the Entrez Gene IDs of genes from the input list. (A) Feasnet based on the raw *p*-values of over-represented REs in the promoter of PKCα (*left*), PKCδ (*middle*) and PKCε (*right*). (B) FDR-adjusted *p*-value based Feasnet of over-represented REs in the promoter of PKCδ-regulated genes. No significantly enriched REs were found in this comparison for either PKCα or PKCε.

A subsequent analysis was carried out to identify REs which are specifically over-represented with a threshold *p*-value≤0.05. Based on the raw *p*-values, the REs over-represented in the PKCδ-regulated genes were AP-1 (129 genes), c-Rel (94 genes), CREB (44 genes), Oct-1 (28 genes), SRF (24 genes), NF-E2 (16 genes), CREBP-1 (16 genes), STAT1 (10 genes), RREB (11 genes), NF-κB (5 genes), transcription factor “R” (3 genes), and Evi-1 (2 genes) **(** **Figure 2A** **)**. Similarly, the over-represented REs for PKCε based on raw *p*-values were Oct-1 (132 genes), c-Rel (57 genes), and HNF-1 (40 genes), and there was in addition over-representation for SRF, CREB, Brn-2, and E2. For PKCα-regulated genes only two REs were over-represented: c-Rel (38 genes) and myogenin (11 genes) REs **(** **Figure 2A** **)**.

Next, we carried out a more stringent analysis of over-represented REs using a false discovery rate (FDR)≤0.30. In the promoter of PKCδ-regulated genes, this analysis revealed enrichment for c-Rel (94 genes), CREB (44 genes), Oct-1 (28 genes), SRF (24 genes), CREBP-1 (16 genes), NF-E2 (16 genes), RREB (11 genes), NF-κB (5 genes), and Evi-1 (2 genes). Thus, c-Rel and CREB represented the most frequent sites in PKCδ-regulated genes **(** **Figure 2B** **)**. However, no significant enrichment in REs was found for either PKCε- or PKCα-regulated genes using this more stringent comparison.

**Figure 3:**
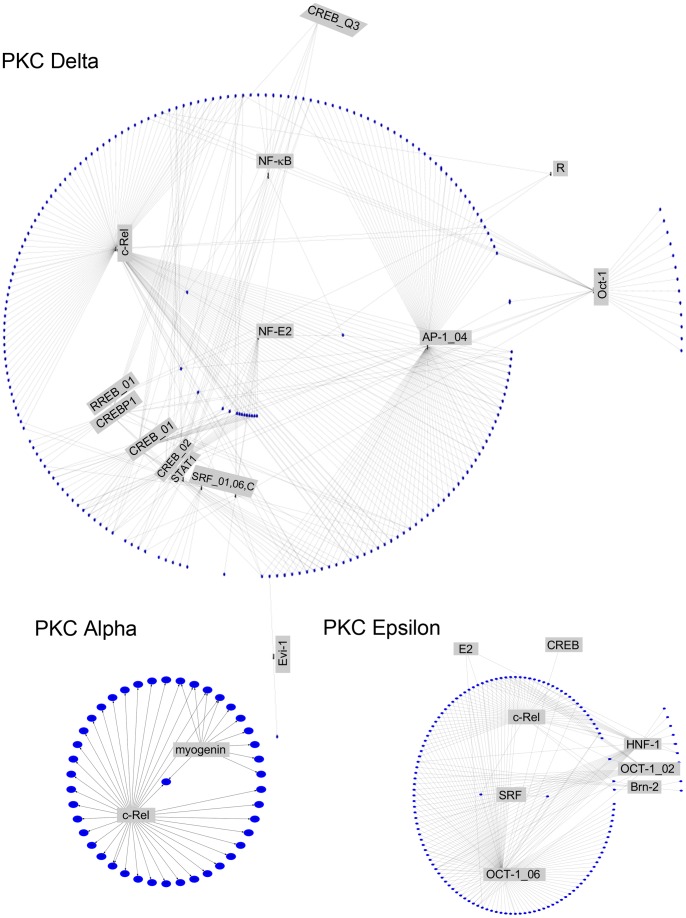
Transcriptional regulatory network diagrams for REs. Transcriptional regulatory network diagrams for REs associated with gene promoters regulated by PKC isozymes, based on the raw *p*-values and derived using GraphViz. *Blue ellipses*, individual genes; *boxes*, REs; *connecting lines*, association between genes and REs as determined using PAINT.

To further visualize the potential connections between the candidate REs over-represented among the genes of each cluster and each of the PKC-regulated genes, network layout diagrams based on the raw *p*-value were generated using GraphViz. These graphic representations revealed the nodes within each network as well as the REs with the potential to coordinately regulate the gene within the cluster. As shown in **Figure 3**, c-Rel displays the highest level of interrelationships, suggesting that this transcription factor may play a prominent role in transcriptional regulation by all PKC isozymes. A second prominent transcription factor is Oct-1, particularly in PKCε-regulated genes and to a minor extent in PKCδ-regulated genes. Based on the FDR (≤0.30)-adjusted *p*-values, network layout diagrams were also generated, which revealed only significant enrichments for PKCδ but not for PKCα or PKCε **(Figure S4)**. Altogether, these results revealed usages of both common and dissimilar REs by each PKC isozyme.

### Validation of PKCδ-regulated Genes in the c-Rel and CREB Transcriptional Networks

**Figure 4:**
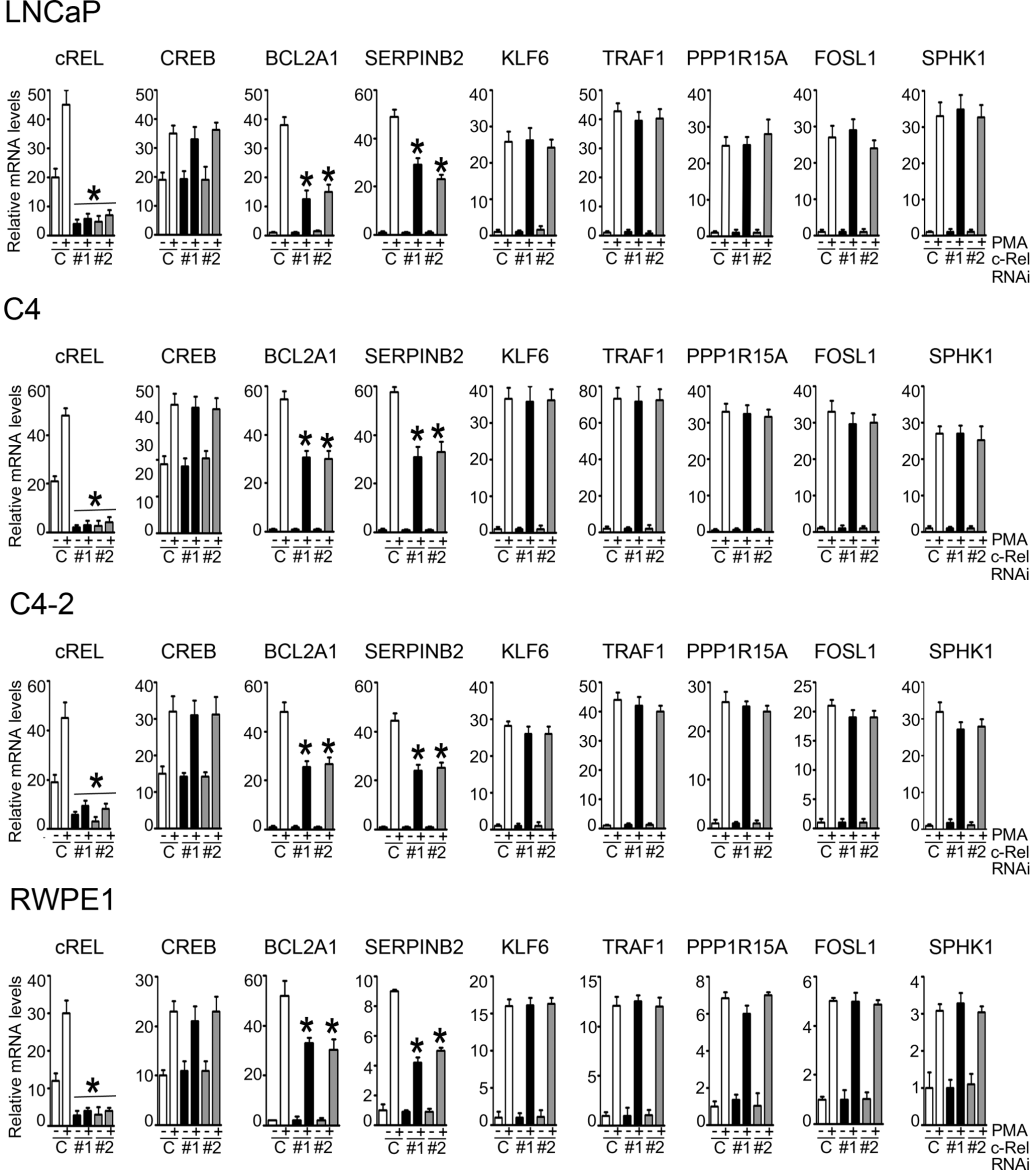
Validation of PKCδ-regulated genes in the c-Rel transcriptional network. c-Rel depletion in LNCaP, C4, C4-2 or RWPE1 cells was achieved using two different RNAi duplexes (#1 and #2). After 48 h, cells were stimulated for 1 h with either 100 nM PMA or vehicle, and mRNA levels of different PKCδ-regulated genes were determined 4 h later using real-time PCR. Results were expressed as fold-increase relative to non-target control RNAi (vehicle-treated). Data represents the mean ± S.E.M. of 3 independent experiments. *C*, non-target control RNAi duplex. *, p<0.05 *vs*. non-target control (PMA-treated).

As PKCδ turned out to be the most prominent isozyme in the control of transcription [15], and due to the relevant roles that this nPKC plays in prostate cancer cells [16], [17], [18], [19], [20], [21], [22], we next focused on the transcription factors identified for PKCδ-regulated genes. As our PAINT analysis established c-Rel and CREB as the major transcriptional regulators, we decided to examine whether they could be implicated in the induction of PKCδ-regulated genes. For this analysis we chose genes filtered/clustered in PAINT as either c-Rel regulated (*BCL2A1* and *SERPINB2),* CREB-regulated (*SERPINB2*, *KLF6, TRAF1* and *PPP1R15A)*, or not regulated by these two transcription factors (*FOSL1* and *SPHK1*), and their expression determined by real-time PCR using specific primers. Several of these PKCδ-regulated genes were previously characterized as important for mediating phorbol ester-induced apoptosis in LNCaP cells [15]. To address this issue, we used RNAi to silence the expression of either c-Rel or CREB in various prostate cellular models (LNCaP, C4, C4-2, and RWPE-1 cells). Various siRNA duplexes at different concentrations were tested (data not shown), and the two most effective were selected in order to minimize the chances of misinterpretation of data due to “off-target” effects. After treatment with PMA the induction of mRNA for the selected PKCδ-regulated genes was determined. As shown in **Figure 4**, silencing c-Rel with two different RNAi duplexes in all four prostate cell lines abrogated c-Rel mRNA expression (both basal and PMA-induced) without affecting the expression of CREB, an indication of the specificity for the knockdown approach. In agreement with our previous study [15], PMA caused a strong induction of the different genes in LNCaP, C4, C4-2, and RWPE-1 cells. Interestingly, c-Rel RNAi depletion specifically reduced PMA-induced expression of *BCL2A1* and *SERPINB2*, without affecting the induction of *KLF6, TRAF1, PPP1R15A*, *FOSL1* or *SPHK1*. Therefore, only those genes identified in the c-Rel transcriptional network through PAINT are sensitive to c-Rel RNAi depletion.

**Figure 5:**
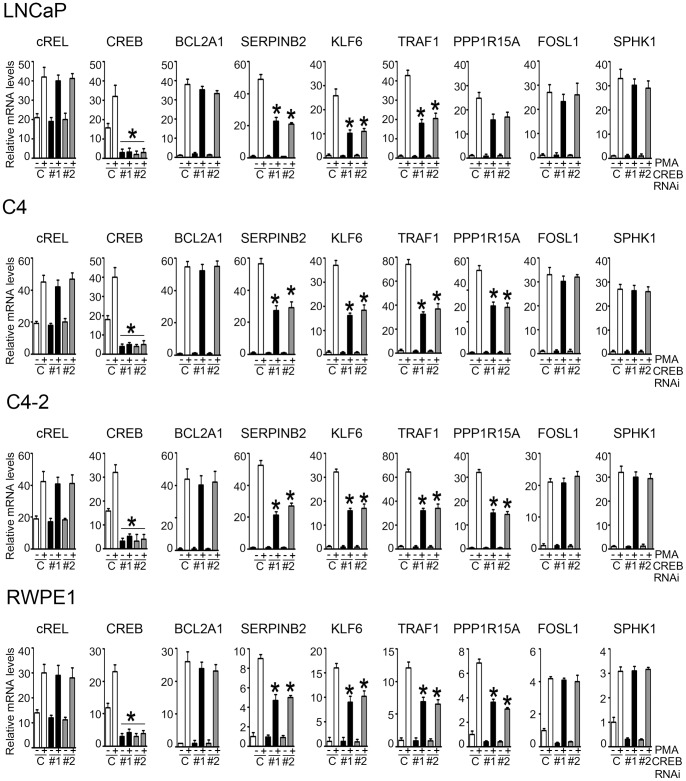
Validation of PKCδ-regulated genes in the CREB transcriptional network. CREB was silenced in LNCaP, C4, C4-2 or RWPE1 cells using two different RNAi duplexes (#1 and #2). After 48 h, cells were stimulated for 1 h with either 100 nM PMA or vehicle, and mRNA levels of different PKCδ-regulated genes were determined 4 h later using real-time PCR. Results were expressed as fold-increase relative to non-target control RNAi (vehicle-treated). Data represents the mean ± S.E.M. of 3 independent experiments. *C*, non-target control RNAi duplex. *, p<0.05 *vs*. non-target control (PMA-treated).

Next, we carried out a similar analysis for those genes that were identified through PAINT to be part of the CREB transcriptional network. CREB was efficiently silenced using two different RNAi duplexes, without any significant changes in the expression of c-Rel mRNA levels **(** **Figure 5** **)**. When we determined the induction of PKCδ-regulated genes by PMA using real-time PCR, we found that CREB RNAi significantly diminished the induction of *SERPINB2*, *KLF6, TRAF1,* and *PPP1R15A*, without affecting the induction of *BCL2A1*, *FOSL1* or *SPHK1*. Thus, only those genes identified in the CREB transcriptional network through PAINT were sensitive to CREB RNAi depletion. This analysis not only validated results from the transcriptional network analysis but also established a distinctive modulation of PKCδ-regulated genes by discrete transcription factors.

**Figure 6:**
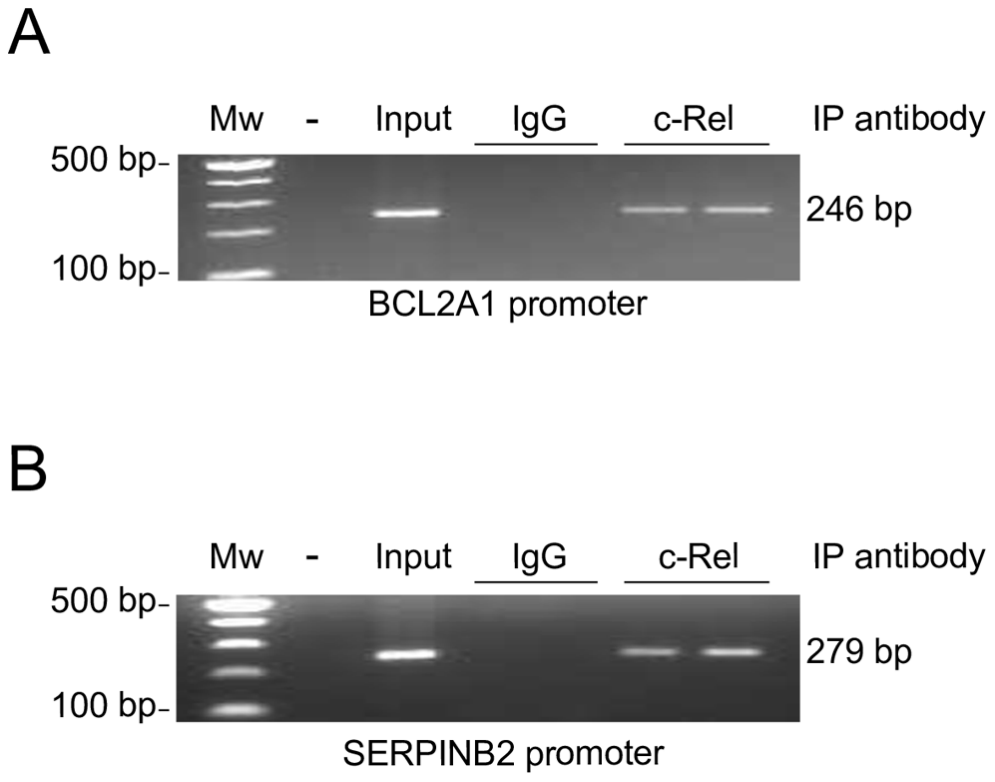
ChIP analysis for c-Rel in PKCδ-regulated genes used for validation. ChIP analysis was performed in LNCaP cells using an anti-c-Rel antibody, and IgG as a negative control. PCR primers were designed to flank known c-Rel binding sites in the BCL2A1 and SERPINB2 promoters. *Mw*, molecular weight marker. Two independent samples for c-Rel and IgG were run in the gel. Similar results were observed in three separate experiments.

Next, we carried out experiments to determine whether c-Rel interacts with promoters of genes identified in our analysis of PKCδ-regulated genes. Using a chromatin immunoprecipitation (ChIP) assay, we found that c-Rel associates with the promoter of *BCL2A1* and *SERPINB2*, the two genes validated in our analysis **(** **Figure 6** **)**, thus confirming direct binding of c-Rel to the promoter of these PKCδ-regulated genes and ruling out the possibility of secondary effects via other transcription factors.

### Functional Gene Categorization of PKCδ/c-Rel-regulated Genes

**Figure 7:**
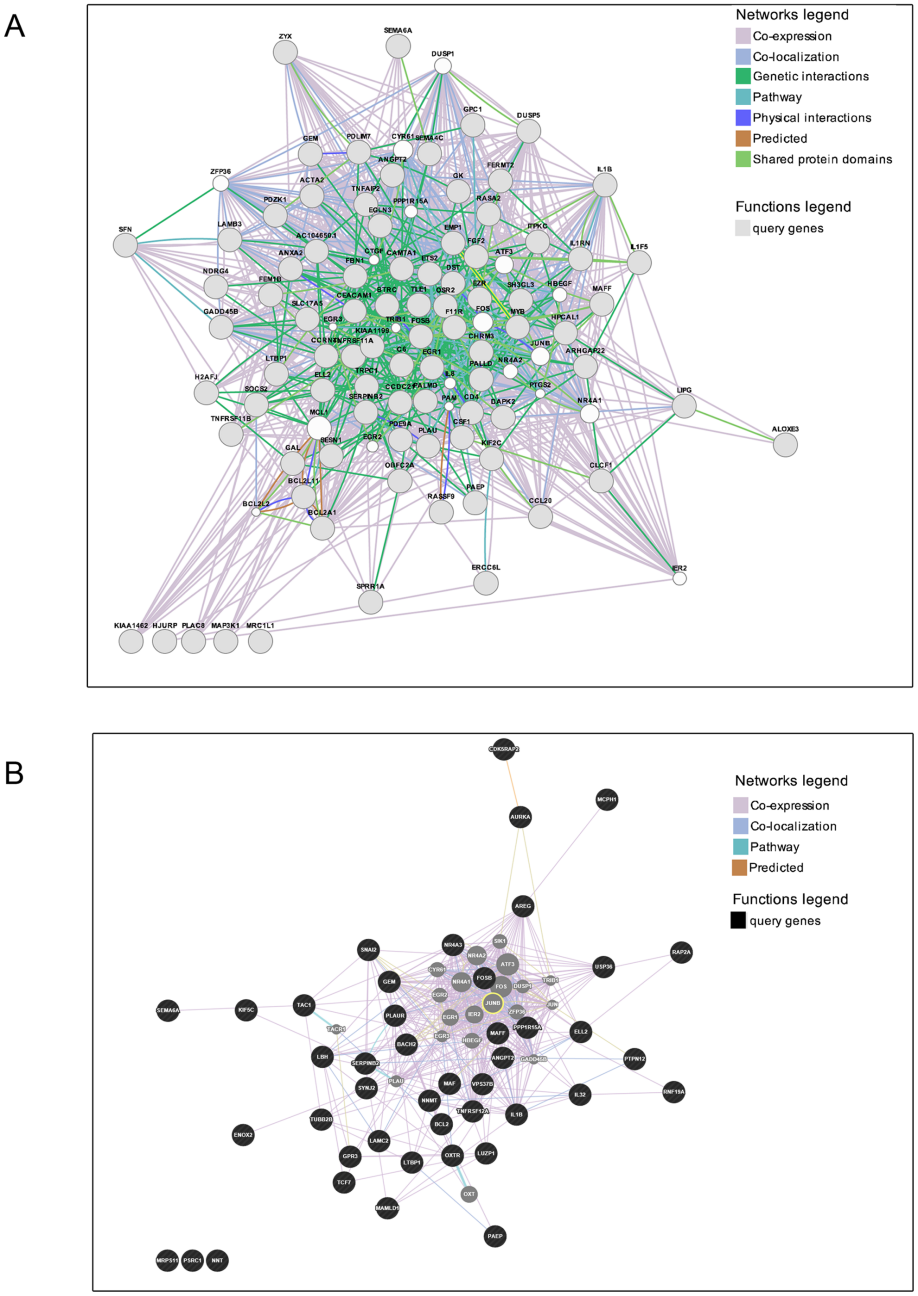
Analysis of functionally related gene networks regulated by c-Rel and CREB. (A) The panel denotes the GeneMANIA-inferred network of PKCδ-regulated genes with c-Rel REs. Query genes to GeneMANIA are marked in *grey* and genes without expression data (absent from the probe set) are in *white*. (B) The panel denotes the GeneMANIA-inferred network of CREB transcriptionally regulated genes. Query genes to GeneMANIA are marked in *black* and genes without expression data (absent from the probe set) are in *grey*.

In order to gain further insight into the possible functional relationships among the PKCδ-regulated genes transcriptionally regulated by c-Rel, we used GeneMANIA (http://www.genemania.org). This analysis would predict the associations in terms of genetic interactions, physical interactions, co-expression, co-localization, shared-protein domains and other predicted parameters. Notably, GeneMANIA revealed that among the 94 PKCδ-regulated genes with c-Rel REs, 89 were found to be part of a functional network **(** **Figure 7A** **)**. Gene Ontology analysis using a FDR <0.05 revealed a strong association of these genes with angiogenesis, inflammatory response, cell migration, cytokine receptor binding, and acute phase response, as specified in Table 1. On the other hand, analysis of the 44 PKCδ-regulated genes with CREB REs, revealed a loose functional association **(** **Figure 7B** **)**. Moreover, unlike the c-Rel-regulated genes, Gene Ontology analysis (FDR<0.05) did not give any relevant biological function associated with CREB-regulated genes. Altogether, these results identified c-Rel as the most prominent transcription factor involved in the control of PKCδ-regulated genes.

**Table 1:** GeneMANIA analysis of c-Rel transcriptionally regulated genes.

GO annotation	FDR	Coverage
query genes	n/a	n/a
Blood vessel endothelial cell migration	8.97E-03	5/38
Regulation of cell migration	8.97E-03	9/216
Vasculature development	8.97E-03	9/247
Acute-phase response	8.97E-03	4/21
Angiogenesis	8.97E-03	8/178
Regulation of cellular component movement	8.97E-03	9/243
Blood vessel development	8.97E-03	9/229
Cell migration involved in sprouting angiogenesis	8.97E-03	4/15
Regulation of cell motility	8.97E-03	9/230
Regulation of locomotion	8.97E-03	9/245
Cytokine receptor binding	8.97E-03	7/119
Sprouting angiogenesis	1.49E-02	4/26
Blood vessel morphogenesis	1.49E-02	8/211
Cytokine activity	2.73E-02	5/64
Positive regulation of homeostatic process	3.17E-02	4/33
Maintenance of location	3.29E-02	6/114
Temperature homeostasis	3.30E-02	1/4
Regulation of response to external stimulus	3.63E-02	7/177
Positive regulation of acute inflammatory response	3.63E-02	3/13
Endothelial cell migration	3.63E-02	5/73
Positive regulation of lipid transport	4.38E-02	3/14
Acute inflammatory response	4.69E-02	4/40
Positive regulation of cell migration	5.45E-02	6/133
Positive regulation of cell motility	5.91E-02	6/136
Regulation of MAPKKK cascade	6.25E-02	7/201
Positive regulation of locomotion	6.67E-02	6/141
Positive regulation of cellular component movement	6.68E-02	6/142
Inflammatory response	9.79E-02	7/220

GeneMANIA analysis for correlation of Gene Ontology annotations with expression status of c-Rel transcriptionally regulated genes. The gene sets are presented with their associated FDR. *Coverage* represents the number of genes present in the network over the total number of genes annotated for that Gene Ontology.

### Loss of c-Rel Sensitizes Prostate Cancer Cells to PMA-induced Apoptosis

**Figure 8:**
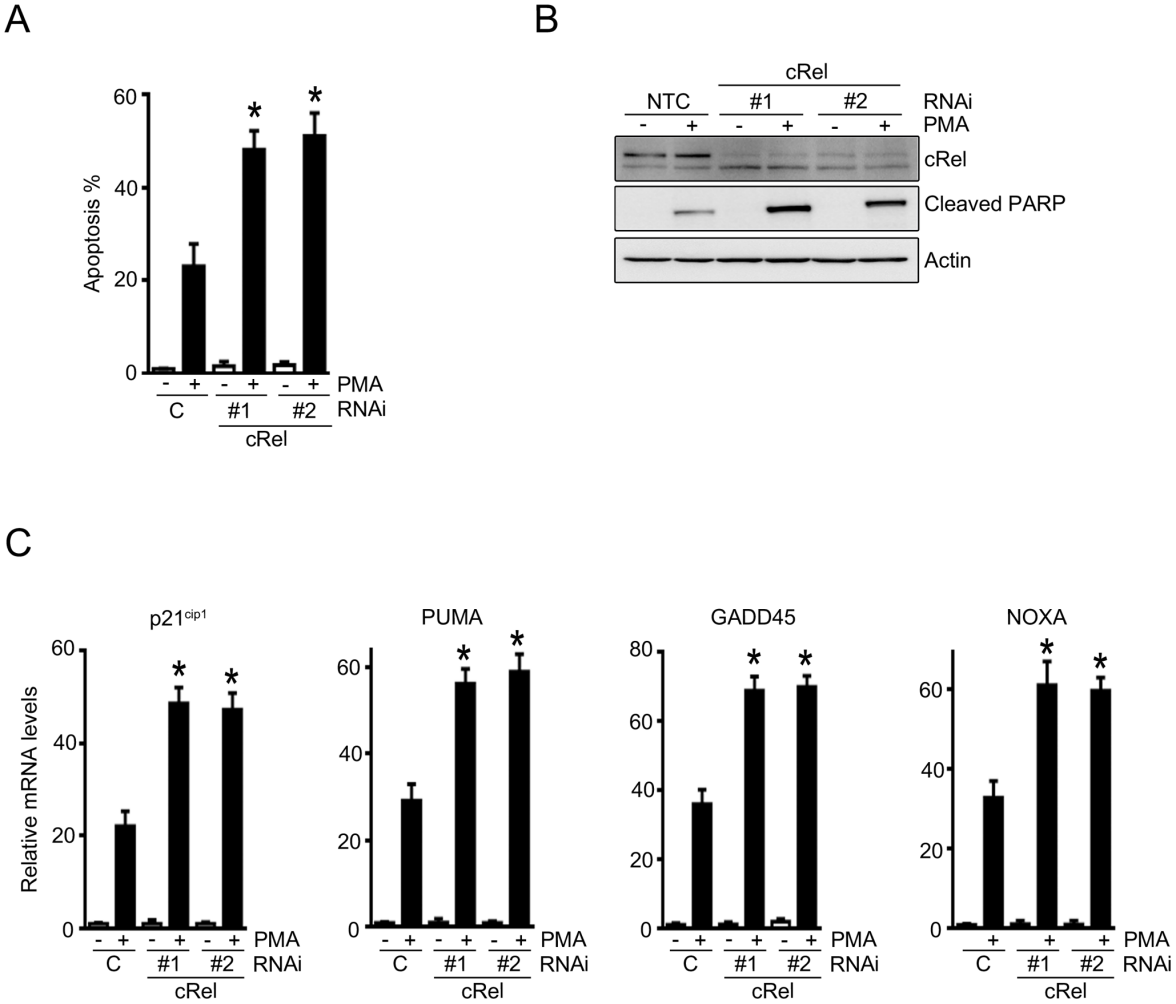
Effect of c-Rel RNAi depletion on PMA-mediated apoptosis. LNCaP cells were transiently transfected with two different c-Rel RNAi duplexes (#1 and #2) or a non-target control (*C*) RNAi duplex. After 24 h, cells were treated with PMA (100 nM, 1 h) or vehicle. (A) Cells were collected after 24 h and stained with DAPI. Incidence of apoptosis in each preparation was analyzed by counting 300 cells and determining the percentage of apoptotic cells. Results were expressed as mean ± S.E.M. of three independent experiments. *, p<0.05 *vs*. control (PMA-treated). (B) Floating and attached cells were collected after 24 h and cell lysates were prepared. Protein expression levels of c-Rel and cleaved PARP were determined by Western blot. Two additional experiments yielded similar results. (C) mRNA expression levels for *p21^cip1^*, *PUMA*, *GADD45*, and *NOXA* were determined 24 h after PMA treatment using real-time PCR. Results were expressed as fold-increase relative to non-target control RNAi (vehicle-treated). Data represents the mean ± S.E.M. of 3 independent experiments. *, p<0.05 *vs*. non-target control RNAi (PMA-treated).

It is established that c-Rel modulates the expression of a number of genes related to apoptosis and survival [23]. Our analysis shows that c-Rel is a key transcriptional modulator of genes regulated by multiple PKC isozymes, which are known to play distinct roles in cell death and survival [4], [15], [18]. We have previously established that PMA activates both apoptotic and survival machineries in androgen-dependent prostate cancer cells [15], [24], [25], [26], however the balance is shifted towards apoptosis [17], [26], [27]. To determine the overall effect of c-Rel on LNCaP cell fate, we used RNAi approach. We observed that knocking down c-Rel in LNCaP cells using two different duplexes significantly enhanced PMA-induced apoptosis **(** **Figure 8A** **)**. We also noticed enhanced PMA-induced PARP cleavage in c-Rel-depleted cells **(** **Figure 8B** **)**. Moreover, c-Rel RNAi increased the expression of c-Rel effector genes known to be involved in growth arrest and apoptosis of prostate cancer cells, such as *p21^cip1^*, *PUMA*, *GADD45* and *NOXA* [28], [29] **(** **Figure 8C** **)**. Therefore, c-Rel drives a pro-survival transcriptional program in LNCaP prostate cancer cells.

## Discussion

Besides their many cellular effects via direct phosphorylation of proteins, PKC isozymes have been also recognized as signaling kinases that trigger significant effects on gene expression upon their activation. The early identification of TREs as *cis*-acting gene promoter elements, together with the well-documented ability of phorbol esters to modulate transcription factor expression/activity, attest to the relevance of PKCs as regulators of transcriptional activation. Moreover, PKC isozymes have been extensively associated with the activation of signaling pathways that modulate gene expression, such as the MEK/Erk, JNK, and NF-κB cascades, just to name a few [5], [8], [12], [24], [26], [30], [31], [32]. In a previous genome-wide analysis of PKC-regulated genes in prostate cancer cell models [15], our laboratory reported marked differences in the ability of DAG/phorbol ester-responsive PKCs α, δ, and ε to regulate gene expression. As anticipated from their distinctive ability to phosphorylate intracellular substrates and activate multiple signaling cascades, PKC isozymes exhibit both overlapping and selective roles in controlling the transcriptional activation of genes. In that study PKCδ was identified as the main PKC isozyme that mediates the induction of genes by phorbol esters, the widely used activators of cPKCs and nPKCs. Indeed, a sizeable number of genes were regulated by this kinase in a specific manner without significant contribution of other DAG/phorbol ester responsive PKCs. In agreement with these results, a recent interesting study also established a prominent role for PKCδ in the control of gene expression in dermal fibroblasts [33]. Among the PKCδ-regulated genes in prostate cancer cells, we identified *FOSL1*, *BCL2A1*, *SERPINB2*, and *TRAF1*. Both *FOSL1* and *BCL2A1* were found to be mediators of phorbol ester- and etoposide-induced apoptosis in LNCaP prostate cancer cells [15]. On a smaller scale, PKCε (and even less PKCα) also controls the transcriptional activation of genes. Accordingly, here we report an exceptionally complex regulation of transcriptional networks by individual members of the PKC family, which may ultimately lead to both selective and overlapping effects of PKC isozymes on gene expression.

In this study we took advantage of the large microarray data generated in our previous report [15] to search for transcription factor binding sites in the promoter regions (up to 5000 bp) of PKCα-, PKCδ-, or PKCε-regulated genes. This analysis revealed a number of REs that are statistically over-represented in the promoters of genes regulated by each of these PKCs as compared to their occurrence with the larger background set of promoters. As expected from the prominent involvement of PKCδ in the regulation of gene expression, there is a higher occurrence frequency of REs for PKCδ-regulated genes relative to PKCε- or PKCα-regulated genes. One of the most notable findings in this analysis is the over-representation of REs for the transcription factor c-Rel in the promoter of genes regulated by these three PKCs. c-Rel is a member of the NF-κB family of dimeric transcription factors that also includes RelA (p65), RelB, p50 and p52, and it binds to the consensus sequence GGGCTTTCC in gene promoters [34], [35]. Alternative c-Rel consensus binding sites have been reported [36], however our analysis using PAINT did not reveal significant enrichment for those motifs. c-Rel controls a myriad of normal cellular functions and development. In addition to its well-characterized role in the immune response and lymphoid malignancies, several studies have linked c-Rel to the progression of epithelial cancers, including prostate, breast, and head and neck cancer [23], [37], [38], [39]. c-Rel displays an abnormally high nuclear expression in cancer and controls the expression of important cell cycle genes [23], [40], [41]. In addition, c-Rel was identified as an important regulator of the DNA damage checkpoint response [42]. Coincidentally, studies from several laboratories have identified PKCs as mediators of cell death and survival. For example, PKCδ mediates apoptosis induced by ara-C, cisplatin, and etoposide, including in prostate cancer cell models such as LNCaP cells [15], [18], [43], [44], [45]. On the other hand, PKCε is a pro-survival kinase in LNCaP cells [4], [24]. Our results suggest that overall, c-Rel drives a pro-survival transcriptional program.

Our analysis also argues for the potential involvement of c-Rel in cellular functions associated with PKCδ. Gene categorization using GeneMANIA revealed that 89 out of the 94 c-Rel/PKCδ transcriptionally regulated genes are part of a functional network that includes genes relevant in inflammation, cytokine responses, angiogenesis, and migration. This is not unexpected, as it agrees with previous roles for PKCδ as a mediator of all these responses. For example, PKCδ has been linked to inflammatory responses and mediates the release of inflammatory cytokines from numerous cell types, and in addition it has been implicated in inflammation-associated neoplastic transformation of epithelial cancers [27], [46], [47]. Previous reports from our laboratory also established that PKCδ mediates the release of TNFα, a pro-inflammatory cytokine involved in prostate cancer progression [25], [27]. Furthermore, TNFα gene induction and responses are mediated by NF-κB transcription factors including c-Rel [48], [49]. PKCδ also mediates migratory responses, such as motility and invasiveness downstream of the EGF receptor or oncogenic stimuli in prostate cancer cells [22], [50].

In summary, this study identified striking differences in the regulation of transcriptional networks by individual members of the PKC family. It is clear that each PKC differently influences gene expression, possibly reflecting their distinct ability to interconnect with signaling cascades that impact on the transcriptional activation of genes. Moreover, the identification of c-Rel as a central hub in the gene network that controls the expression of PKC-regulated genes underscores a novel functional link that may have significant implications in inflammation and cancer. Not surprisingly, a recent study revealed that pharmacological inhibition of PKCδ leads to profound changes in the transcriptome of fibroblasts from patients with scleroderma, a disease linked with inflammation, with NF-κB transcription factors playing a central role in the control of those genes [33]. One may predict that despite the obvious disparities that may be observed in different cellular models, the PKC-NF-κB interconnection could be potentially exploited for a number of therapeutic purposes.

## Materials and Methods

### Cell Culture and Reagents

LNCaP (ATCC), C4 and C4-2 [51] human prostate cancer cells were cultured in RPMI 1640 medium supplemented with 10% FBS, penicillin (100 U/ml) and streptomycin (100 µg/ml) at 37°C in a humidified 5% CO_2_ atmosphere, as previously described [15]. Human normal immortalized prostate epithelial RWPE-1 cells (ATCC) were cultured as previously described [52]. Phorbol 12-myristate 13-acetate (PMA) was obtained from LC Laboratories (Woburn, MA). Fetal bovine serum was purchased from Hyclone (Logan, UT). Other cell culture reagents and media were from ATCC (Rockville, MD).

### RNA Interference (RNAi)

LNCaP cells were transfected with different siRNAs (120 pmol) using the Amaxa Nucleofector (Amaxa Biosystems, Gaithersburg, MD) and 48 h later used for the indicated experiments. For transient depletion we used ON-TARGET Plus RNAi duplexes purchased from Dharmacon (Lafayette, CO). We used two different RNAi sequences in each case. PKC isozyme RNAi sequences for our microarray study are described elsewhere [15], [16]. Other customized target sequences were as follows: CREB RNAi #1: GAGAGAGGUCCGUCUAAUG; CREB RNAi #2: UAGUACAGCUGCCCAAUGG; c-Rel RNAi #1: GAGCACAGCACAGACAACAACCGAA; c-Rel RNAi #2: CCGUGCUCCAAAUACUGCAGAAUUA. As a non-target control RNAi we used the Control Negative Silencer® from Ambion (Austin, TX).

### Computational Analysis of Transcriptional Regulatory Networks

In order to identify gene regulatory regions in the promoters of PKC-regulated genes we used the Promoter Analysis and Interaction Network Toolset (PAINT) v4.0 available at the Daniel Baugh Institute homepage (http://www.dbi.tju.edu/dbi/tools/paint). Three groups of genes regulated by PKCα, PKCδ or PKCε from our previously published microarray data [15] were used. Probesets were filtered as follows: *i*) genes that were differentially expressed in response to PMA treatment by at least a factor of 2 (*i.e.* −2≤PMA/vehicle≤2); and *ii*) fold-change by PMA was either reduced by ≥ 50% or augmented by ≥ 50% as a consequence of PKC isozyme RNAi depletion (see Tables S1, S2, and S3). Transcription response elements (REs) were identified in predicted promoter sequences (5000 bp region upstream of the transcription start site), with inclusion of the complimentary strand analysis and 1.0 core similarity threshold [53], [54]. The TRANSFAC Public v7.0 database (www.gene-regulation.com) was used by PAINT to predict known REs. These REs were entered into the Feasibility Network Builder module of PAINT (Feasnet Builder), which constructed a candidate interaction matrix (CIM) that depicts a graphic representation of their occurrence within the gene set. Enrichment analysis was then performed using PAINT to compute *p*-values indicating either over- or under-representation of REs within the selected gene respect to the reference (Human Genome U133A_2.0 Array). *p*-values were calculated using the hypergeometric distribution and adjusted for multiple testing using false discovery rate (FDR). Multiple visualizations (Feasnet, Graphic Network) for these analyses were obtained.

### Gene Functional Network Inferences

To establish functional interactions, subsets of PKCδ-regulated genes that have been categorized under the transcriptional control of either c-Rel or CREB were analyzed using GeneMANIA (http://www.genemania.org). Analysis of results was conducted using the default network weighting method. A collection of Gene Ontology gene sets was used to test for the correlation with the expression status of c-Rel- and CREB-transcriptionally regulated genes.

### RNA Isolation and cDNA Synthesis

Subconfluent cells were treated for 1 h with either 100 nM PMA or vehicle. Four hours later RNA was extracted using the RNeasy kit (Qiagen, Valencia, CA). Two µg of RNA per sample were reverse transcribed using random hexamers as primers and the Taqman reverse transcription reagent kit (Applied Biosystems, Branchburg, NJ). Each sample was analyzed in triplicate by real-time PCR. Experiments were repeated three times.

### Real-time PCR

PCR primers and fluorogenic probes for BCL2A1, KLF6, SERPINB2, TRAF1, PPP1R15A, SPHK1, FOSL1, c-REL and CREB were purchased from Applied Biosystems. Probes were 5′end-labeled with 6-carboxyfluorescein (FAM). PCR amplifications were performed using an ABI PRISM 7700 Detection System, as previously reported [15]. Reactions were carried out in a total volume of 12.5 µl containing Taqman Universal PCR Master Mix (Applied Biosystems), commercial target primers (300 nM), the fluorescent probe (200 nM), and 1 µl of cDNA. PCR product formation was continuously monitored using the Sequence Detection System software version 1.7 (Applied Biosystems). The FAM signal was normalized to endogenous 18S ribosomal RNA.

### Western Blots

Western blot analysis was carried out essentially as previously described [16]. Bands were visualized by the Enhanced Chemiluminescence (ECL) Western blotting detection system. Images were captured using a FUJIFILM LAS-3000 and the LAS-2000 software. The following antibodies were used: anti-c-Rel (1∶1,000, Santa Cruz Biotechnology Inc., Santa Cruz, CA); anti-cleaved PARP (1∶1000, Cell Signaling Technology Inc., Danvers, MA); and anti-β-actin (1∶50,000, Sigma-Aldrich, St. Louis, MO). Anti-mouse or anti-rabbit secondary antibodies conjugated to horseradish peroxidase (1∶5000, Bio-Rad Laboratories, Hercules, CA) were used.

### Apoptosis Assays

The incidence of apoptosis was determined as we described previously [17]. Briefly, cells were trypsinized, mounted on glass slides, fixed in 70% ethanol, and then stained for 20 min with 1 mg/ml 4, 6- diamidino-2-phenylindole (DAPI). Apoptotic cells were characterized by chromatin condensation and fragmentation when examined by fluorescence microscopy. The incidence of apoptosis was analyzed by counting 300 cells in each preparation.

### Chromatin Immunoprecipitation Assay

ChIP assay was performed as described previously [55] with slight modifications. Briefly, LNCaP cells (3×10^6^) were fixed in 1% formaldehyde for 15 min to cross-link DNA with associated proteins. The reaction was stopped by the addition of 125 mM glycine buffer. Cells were collected and lysed in a buffer containing 50 mM Tris-HCl, pH 8.1, 1% SDS, 10 mM EDTA, and protease and phosphatase inhibitors. Lysates were sonicated, and equal amounts of chromatin were diluted in ChIP buffer (16.7 mM Tris-HCl, pH 8.1, 0.01% SDS, 1.1% Triton X-100, 1.2 mM EDTA, and 167 mM NaCl). As an input, we used 10% of the sample. To reduce non-specific background, the chromatin solution was pre-cleared with protein A agarose/salmon sperm DNA and then incubated overnight at 4°C with an anti-c-Rel antibody (5 mg, Santa Cruz Biotechnology) or a rabbit IgG as control, followed by 1 h of incubation with protein A agarose/salmon sperm DNA beads. Samples were centrifuged, and the pellets sequentially washed with low salt buffer (20 mM Tris-HCl, pH 8.1, 0.1% SDS, 1% Triton X-100, 2 mM EDTA, 150 mM NaCl); high salt buffer (20 mM Tris-HCl, pH 8.1, 0.1% SDS, 1% Triton X-100, 2 mM EDTA, 500 mM NaCl); LiCl wash buffer (10 mM Tris-HCl, pH 8.1, 0.25 M LiCl, 1% Nonidet P-40, 1% deoxycholate, 1 mM EDTA) and TE buffer (10 mM Tris-HCl, pH 8.0, 1 mM EDTA). Protein-DNA complexes were eluted in a buffer containing 1% SDS and 0.1 M NaHCO_3_. The cross-linking was reversed by incubation in 200 mM NaCl overnight at 65°C followed by proteinase K digestion in 40 mM Tris-HCl, pH 6.5, 10 mM EDTA (1 h at 55°C). DNA was recovered by phenol/chloroform extraction and ethanol precipitation and subsequently analyzed by PCR. Primers for *c-Rel* were as follows: forward, 5′-GCCTCAAATTGACCGGACT; reverse, 5′-CAGTGCTTTCCAAGCTGTCA (human *BCL2A1* promoter); and forward, 5′- AGGGTGACACTCCAGATTGC; reverse, 5′- TCTGCTTGGAAGGGAACCTA (human *SERPINB2* promoter).

### Statistical Analysis

Results were compared by analysis of variance using GraphPad Prism 5.0. In all cases, p<0.05 was considered statistically significant.

## Supporting Information

Figure S1 **Complete candidate interaction (CIM) matrix for REs in PKCα-regulated genes, as determined from PAINT analysis.** *Columns* correspond to the TRANSFAC identifiers for each over-represented RE. *Rows* represent the genes from the input list with their corresponding Entrez Gene IDs. REs listed along the x-axis are clustered according to related occurrence pattern (19). The elements within the matrix are color-coded based upon the *p*-value obtained for each RE found in the regulatory regions of the genes (5000 bp). *Red boxes*, over-represented REs (p<0.05); *blue boxes*, under-represented REs (p<0.05); *grey boxes*, REs in the gene list with no statistical significance. *Note:* this is a large-format figure and should be viewed at enhanced magnification.
(PDF)

Figure S2 **Complete candidate interaction matrix for REs in PKCδ-regulated genes, as determined from PAINT analysis.** *Columns* correspond to the TRANSFAC identifiers for each over-represented RE. *Rows* represent the genes from the input list with their corresponding Entrez Gene IDs. REs listed along the x-axis are clustered according to related occurrence pattern (19). The elements within the matrix are color-coded based upon the *p*-value obtained for each RE found in the regulatory regions of the genes (5000 bp). *Red boxes*, over-represented REs (p<0.05); *blue boxes*, under-represented REs (p<0.05); *grey boxes*, REs in the gene list with no statistical significance. *Note:* this is a large-format figure and should be viewed at enhanced magnification.
(PDF)

Figure S3 **Complete candidate interaction matrix for REs in PKCε-regulated genes, as determined from PAINT analysis.** *Columns* correspond to the TRANSFAC identifiers for each over-represented RE. *Rows* represent the genes from the input list with their corresponding Entrez Gene IDs. REs listed along the x-axis are clustered according to related occurrence pattern (19). The elements within the matrix are color-coded based upon the *p*-value obtained for each RE found in the regulatory regions of the genes (5000 bp). *Red boxes*, over-represented REs (p<0.05); *blue boxes*, under-represented REs (p<0.05); *grey boxes*, REs in the gene list with no statistical significance. *Note:* this is a large-format figure and should be viewed at enhanced magnification.
(PDF)

Figure S4 **Transcriptional regulatory network diagram for REs associated with promoter regions regulated by PKCδ.** Network visualization of Feasnet based on the FDR (<0.03)-adjusted *p*-values was derived using GraphViz. No significantly enriched REs were found in this comparison for either PKCα or PKCε. *Blue ellipses*, individual genes; *boxes*, REs; *connecting lines*, gene-RE associations.
(PDF)

Table S1 PKCα-regulated genes used for PAINT analysis. Differentially expressed genes identified in our previous microarray analysis (15) were filtered as a) altered in response to PMA by a factor of 2 (−2≤PMA/vehicle≤2); and b) fold-change by PMA is either reduced by ≥ 50% or augmented by ≥ 50% as a consequence of PKCα RNAi depletion.
(DOC)

Table S2 PKCδ-regulated genes used for PAINT analysis. Differentially expressed genes identified in our previous microarray analysis (15) were filtered as: a) altered in response to PMA by a factor of 2 (−2≤PMA/vehicle≤2); and b) fold-change by PMA is either reduced by ≥ 50% or augmented by ≥ 50% as a consequence of PKCδ RNAi depletion.
(DOC)

Table S3 PKCε-regulated genes used for PAINT analysis. Differentially expressed genes identified in our previous microarray analysis (15) were filtered as: a) altered in response to PMA by a factor of 2 (−2≤PMA/vehicle≤2); and b) fold-change by PMA is either reduced by ≥ 50% or augmented by ≥ 50% as a consequence of PKCε RNAi depletion.
(DOC)
