# Supplementary figures and images for: Regulation of Transcriptional Networks by PKC Isozymes: Identification of c-Rel as a Key Transcription Factor for PKC-Regulated Genes
Source: PLoS One. 2013 Jun 27;8(6):e67319. doi: 10.1371/journal.pone.0067319 (PMC3694964; doi:10.1371/journal.pone.0067319)

Figure S1

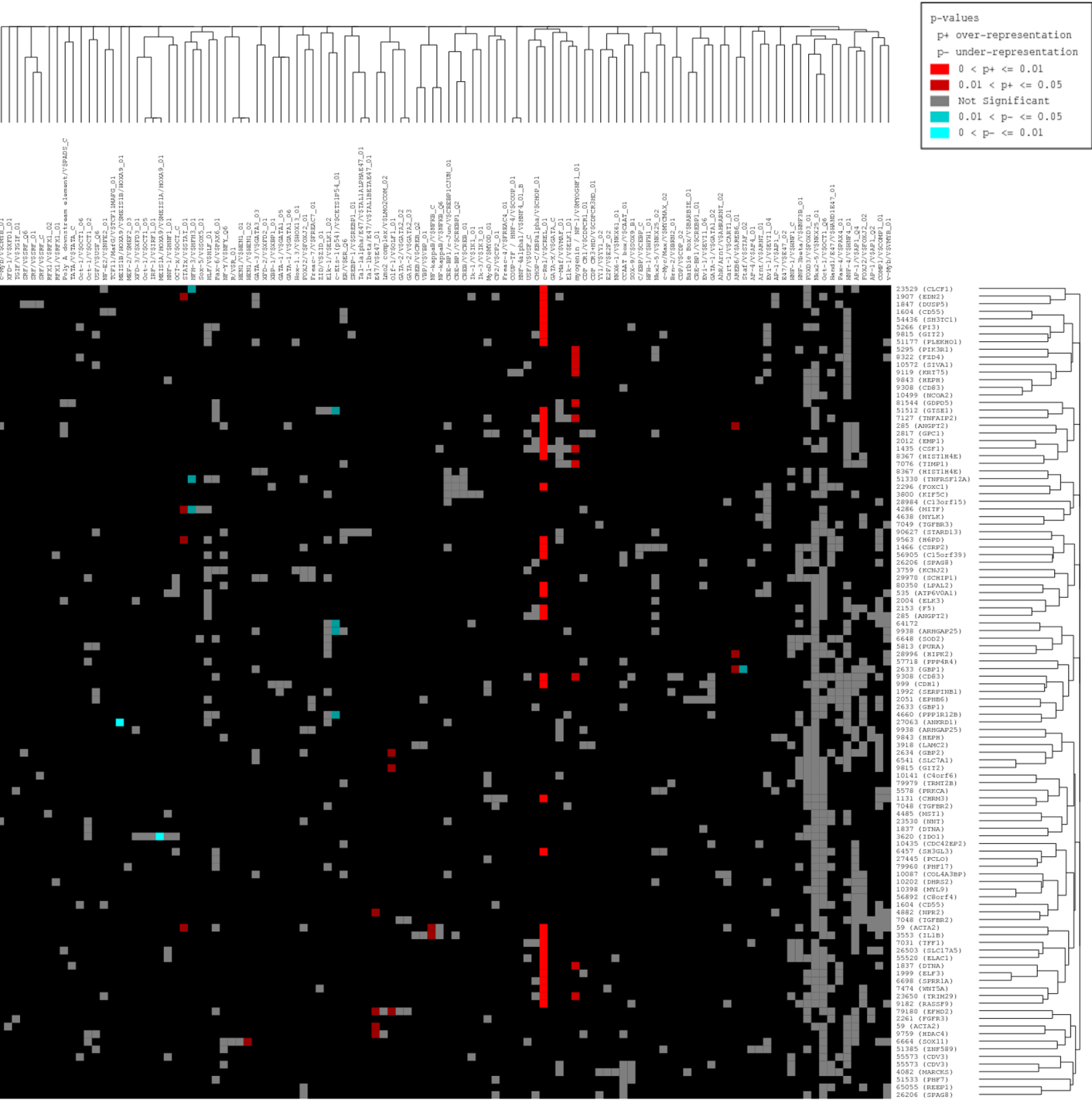

Supplement: Figure S1 — Complete candidate interaction (CIM) matrix for REs in PKCα-regulated genes, as determined from PAINT analysis. Columns correspond to the TRANSFAC identifiers for each over-represented RE. Rows represent the genes from the input list with their corresponding Entrez Gene IDs. REs listed along the x-axis are clustered according to related occurrence pattern (19). The elements within the matrix are color-coded based upon the p-value obtained for each RE found in the regulatory regions of the genes (5000 bp). Red boxes, over-represented REs (p<0.05); blue boxes, under-represented REs (p<0.05); grey boxes, REs in the gene list with no statistical significance. Note: this is a large-format figure and should be viewed at enhanced magnification. (PDF) [file pone.0067319.s001.pdf]

# Figure S2

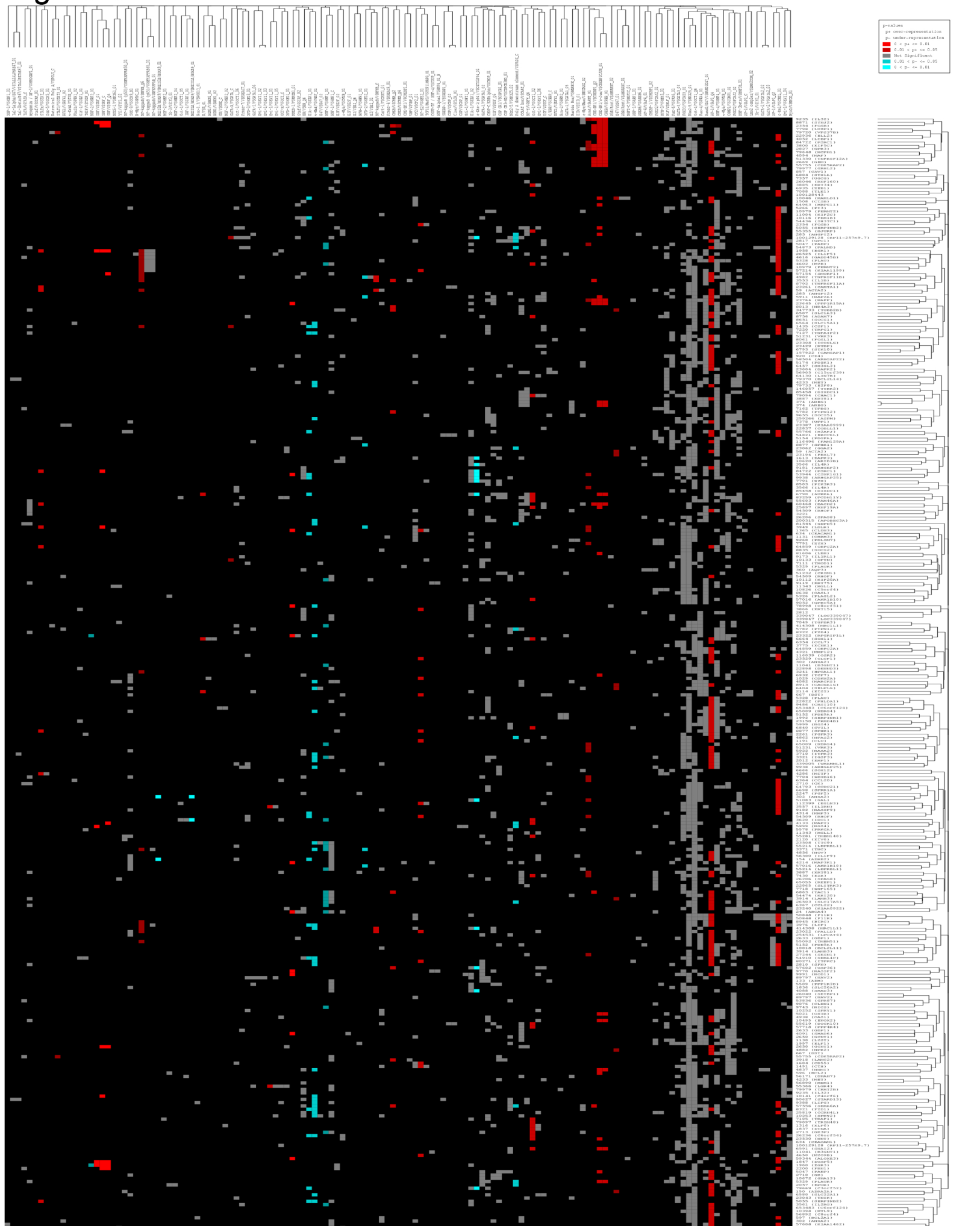

Supplement: Figure S2 — Complete candidate interaction matrix for REs in PKCδ-regulated genes, as determined from PAINT analysis. Columns correspond to the TRANSFAC identifiers for each over-represented RE. Rows represent the genes from the input list with their corresponding Entrez Gene IDs. REs listed along the x-axis are clustered according to related occurrence pattern (19). The elements within the matrix are color-coded based upon the p-value obtained for each RE found in the regulatory regions of the genes (5000 bp). Red boxes, over-represented REs (p<0.05); blue boxes, under-represented REs (p<0.05); grey boxes, REs in the gene list with no statistical significance. Note: this is a large-format figure and should be viewed at enhanced magnification. (PDF) [file pone.0067319.s002.pdf]

# Figure S3

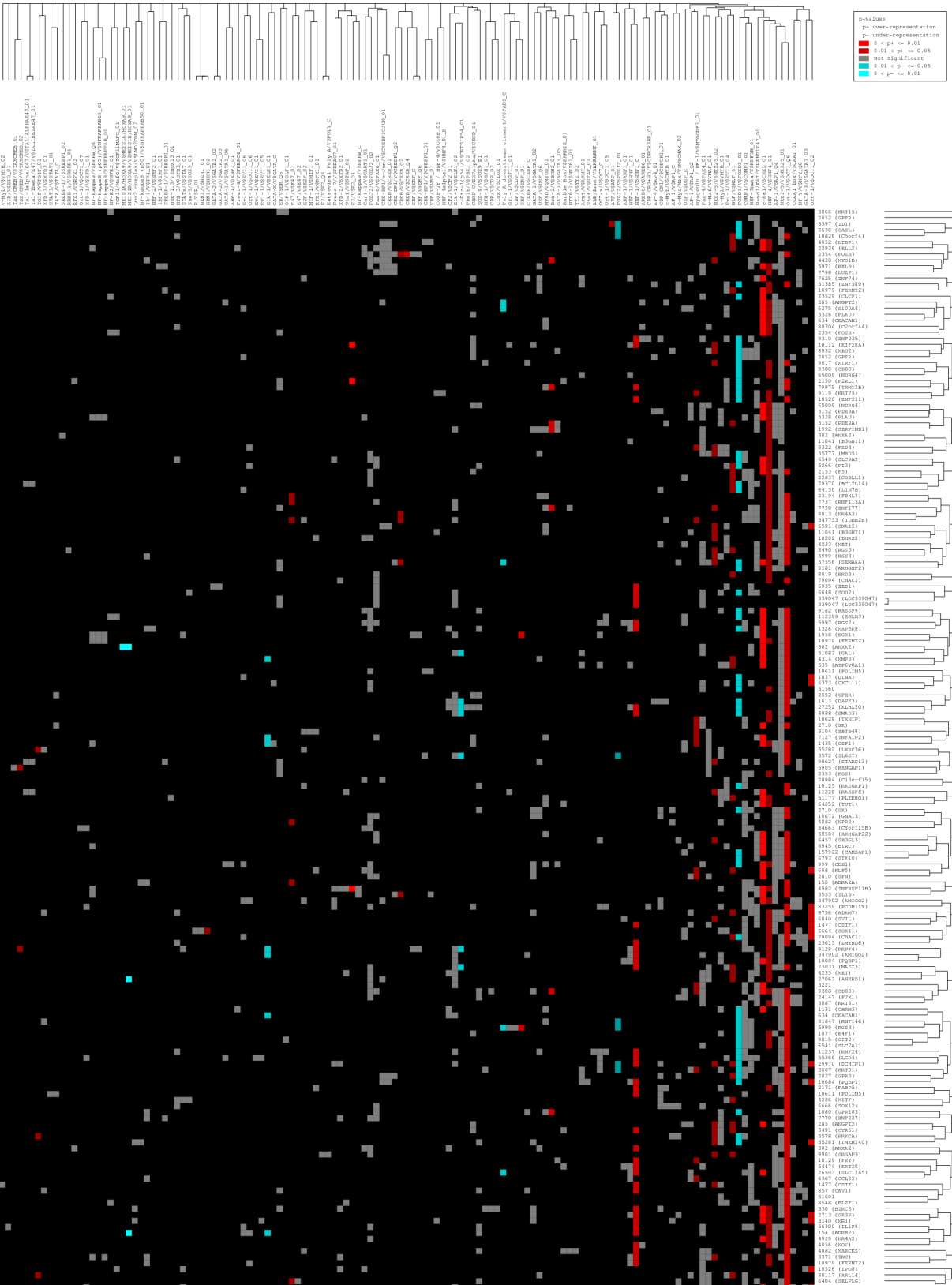

Supplement: Figure S3 — Complete candidate interaction matrix for REs in PKCε-regulated genes, as determined from PAINT analysis. Columns correspond to the TRANSFAC identifiers for each over-represented RE. Rows represent the genes from the input list with their corresponding Entrez Gene IDs. REs listed along the x-axis are clustered according to related occurrence pattern (19). The elements within the matrix are color-coded based upon the p-value obtained for each RE found in the regulatory regions of the genes (5000 bp). Red boxes, over-represented REs (p<0.05); blue boxes, under-represented REs (p<0.05); grey boxes, REs in the gene list with no statistical significance. Note: this is a large-format figure and should be viewed at enhanced magnification. (PDF) [file pone.0067319.s003.pdf]

Figure S4

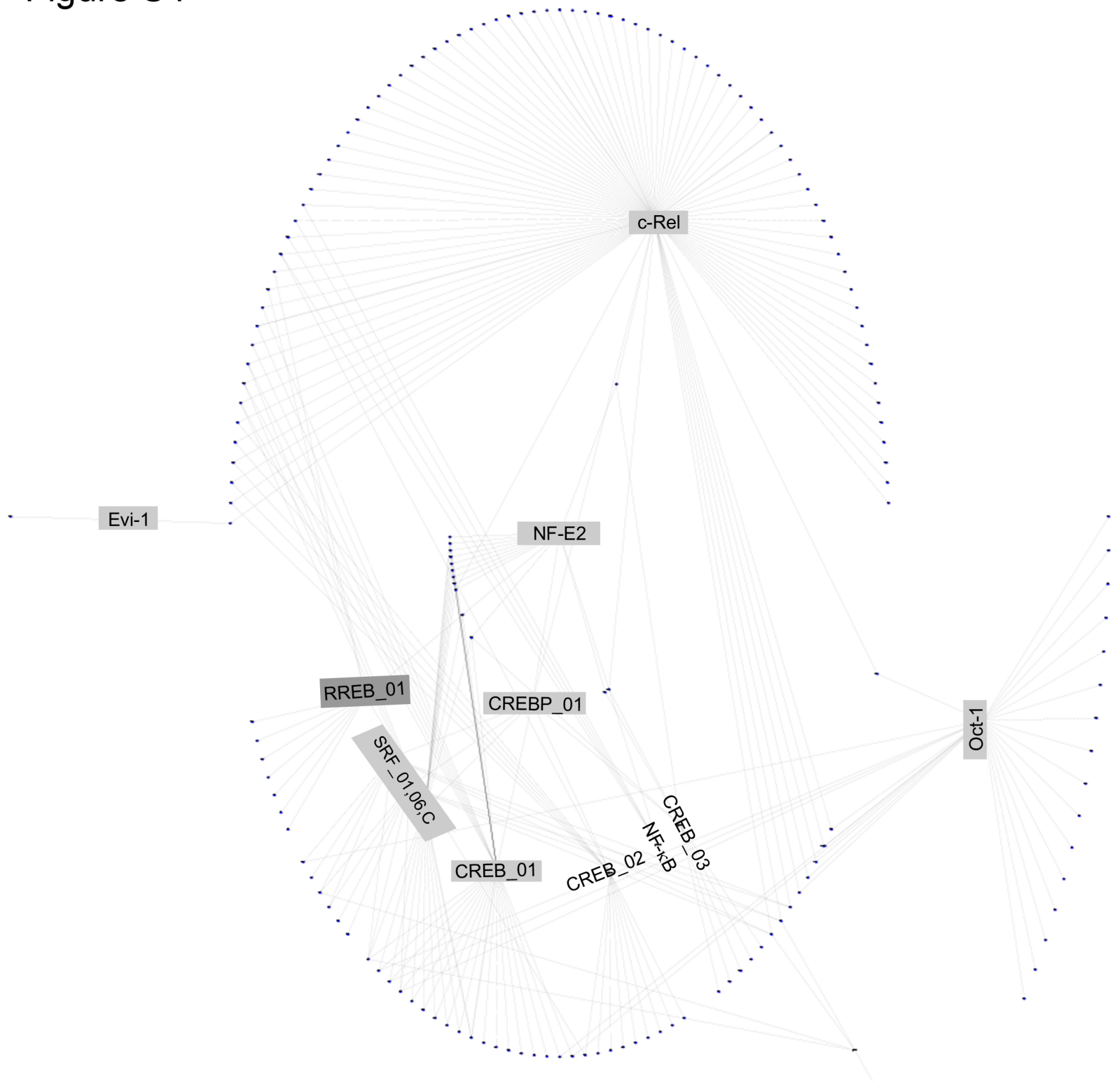

Supplement: Figure S4 — Transcriptional regulatory network diagram for REs associated with promoter regions regulated by PKCδ. Network visualization of Feasnet based on the FDR (<0.03)-adjusted p-values was derived using GraphViz. No significantly enriched REs were found in this comparison for either PKCα or PKCε. Blue ellipses, individual genes; boxes, REs; connecting lines, gene-RE associations. (PDF) [file pone.0067319.s004.pdf]
