# Supplementary material for: Regulation of Transcriptional Networks by PKC Isozymes: Identification of c-Rel as a Key Transcription Factor for PKC-Regulated Genes
Source: PLoS One. 2013 Jun 27;8(6):e67319. doi: 10.1371/journal.pone.0067319 (PMC3694964; doi:10.1371/journal.pone.0067319)
Supplement: Table S1 — PKCα-regulated genes used for PAINT analysis. Differentially expressed genes identified in our previous microarray analysis (15) were filtered as a) altered in response to PMA by a factor of 2 (−2≤PMA/vehicle≤2); and b) fold-change by PMA is either reduced by ≥ 50% or augmented by ≥ 50% as a consequence of PKCα RNAi depletion. (DOC) [file pone.0067319.s005.doc]

**Table S1. PKCα-regulated genes used for PAINT analysis.**

| **Gene Symbol** | **Entrez Gene** | **p-value(PKCalpha * PMA vs. PKCalpha * veh)** | **Fold-Change(PKCalpha * PMA vs. PKCalpha * veh)** |
| --- | --- | --- | --- |
| DUSP5 | 1847 | 9.55E-23 | 362.155 |
| EDN2 | 1907 | 3.60E-34 | 272.405 |
| C8orf4 | 56892 | 1.02E-27 | 165.962 |
| TNFRSF12A | 51330 | 7.10E-24 | 104.958 |
| GBP1 | 2633 | 7.47E-27 | 89.047 |
| PI3 | 5266 | 8.46E-21 | 72.8718 |
| TGFBR2 | 7048 | 1.26E-31 | 63.3059 |
| PI3 | 5266 | 3.55E-22 | 59.4302 |
| PRKCA | 5578 | 1.15E-29 | 38.3829 |
| TFF1 | 7031 | 5.73E-21 | 33.4632 |
| SPAG8 | 26206 | 6.48E-28 | 32.3652 |
| ELF3 | 1999 | 6.36E-23 | 32.0554 |
| CSF1 | 1435 | 4.46E-20 | 29.9782 |
| GPC1 | 2817 | 1.15E-24 | 28.9465 |
| NNT | 23530 | 3.22E-18 | 25.8796 |
| PRKCA | 5578 | 5.01E-23 | 25.7264 |
| SERPINB1 | 1992 | 5.81E-18 | 22.9181 |
| CLCF1 | 23529 | 8.91E-16 | 22.8271 |
| MITF | 4286 | 1.92E-21 | 19.821 |
| NPR2 | 4882 | 1.82E-20 | 19.0287 |
| ANKRD1 | 27063 | 8.24E-15 | 14.0697 |
| SCHIP1 | 29970 | 4.11E-18 | 12.8534 |
| TRIM29 | 23650 | 8.91E-21 | 12.8192 |
| TIMP1 | 7076 | 5.50E-22 | 12.6866 |
| ELK3 | 2004 | 2.96E-15 | 12.346 |
| EPHB6 | 2051 | 2.57E-14 | 11.2095 |
| NPR2 | 4882 | 4.43E-17 | 10.8617 |
| GBP2 | 2634 | 8.97E-21 | 10.7566 |
| LAMC2 | 3918 | 1.08E-14 | 10.5411 |
| MYL9 | 10398 | 2.50E-11 | 10.3175 |
| CSRP2 | 1466 | 3.88E-10 | 9.63595 |
| HEPH | 9843 | 2.54E-18 | 8.65207 |
| ANGPT2 | 285 | 3.63E-07 | 8.46618 |
| C15orf39 | 56905 | 1.21E-11 | 8.01109 |
| ANGPT2 | 285 | 1.52E-09 | 7.85296 |
| CDC42EP2 | 10435 | 6.46E-18 | 7.58405 |
| C13orf15 | 28984 | 1.54E-11 | 7.47751 |
| CD55 | 1604 | 1.45E-13 | 6.92147 |
| TGFBR3 | 7049 | 1.05E-06 | 5.97927 |
| CD24 | 934 | 4.64E-10 | 5.85592 |
| SH3TC1 | 54436 | 1.72E-17 | 5.85442 |
| SOD2 | 6648 | 4.42E-07 | 5.75972 |
| KIAA1622 | 57718 | 6.22E-12 | 5.57245 |
| IL1B | 3553 | 2.20E-09 | 5.13738 |
| C15orf39 | 56905 | 2.35E-13 | 5.05794 |
| ACTA2 | 59 | 5.74E-10 | 4.94932 |
| EMP1 | 2012 | 2.52E-11 | 4.86504 |
| CD83 | 9308 | 7.94E-07 | 4.36796 |
| IL1B | 3553 | 9.84E-09 | 4.23839 |
| SH3GL3 | 6457 | 1.58E-09 | 4.2356 |
| EFHD2 | 79180 | 4.15E-14 | 4.22584 |
| ARHGAP25 | 9938 | 4.33E-06 | 3.9146 |
| SOX11 | 6664 | 1.95E-07 | 3.86924 |
| CDV3 | 55573 | 2.21E-05 | 3.79737 |
| FOXC1 | 2296 | 2.25E-13 | 3.68605 |
| PLEKHO1 | 51177 | 2.10E-07 | 3.64811 |
| HIPK2 | 28996 | 0.0120733 | 3.26384 |
| RP4-724E16.2 | 80089 | 0.000212343 | 2.92364 |
| MYLK | 4638 | 0.00048438 | 2.86846 |
| TNFAIP2 | 7127 | 0.000238966 | 2.75899 |
| MARCKS | 4082 | 0.0098754 | 2.70873 |
| SLC7A1 | 6541 | 0.0126546 | 2.707 |
| KRT75 | 9119 | 0.0192918 | 2.32974 |
| CDH1 | 999 | 0.00764433 | 2.24939 |
| CHRM3 | 1131 | 0.00533754 | 2.02627 |
| SPRR1A | 6698 | 0.0221093 | 1.65658 |
| ARHGAP25 | 9938 | 0.0271487 | 1.57175 |
| ATP6V0A1 | 535 | 0.0673319 | 1.53293 |
| RASSF9 | 9182 | 0.103416 | 1.34982 |
| SOX11 | 6664 | 0.164967 | 1.34883 |
| DTNA | 1837 | 0.0808506 | 1.29257 |
| INDO | 3620 | 0.122698 | 1.24945 |
| SLC17A5 | 26503 | 0.77379 | 1.2307 |
| GDPD5 | 81544 | 0.14588 | 1.19736 |
| C4orf6 | 10141 | 0.336482 | 1.17777 |
| REEP1 | 65055 | 0.304954 | 1.12463 |
| H6PD | 9563 | 0.977268 | 1.01622 |
| CXorf34 | 79979 | 0.833632 | -1.20877 |
| KIF5C | 3800 | 0.295539 | -1.57758 |
| ELAC1 | 55520 | 1.17E-09 | -3.13389 |
| HIST1H4E | 8367 | 2.21E-08 | -3.2298 |
| F5 | 2153 | 3.31E-07 | -3.32965 |
| PHF7 | 51533 | 2.21E-06 | -3.50764 |
| GIT2 | 9815 | 1.60E-11 | -3.62258 |
| ZNF589 | 51385 | 6.20E-13 | -3.8018 |
| NCOA2 | 10499 | 6.27E-08 | -4.03692 |
| COL4A3BP | 10087 | 1.72E-05 | -4.03924 |
| GTSE1 | 51512 | 1.35E-06 | -4.24973 |
| SIVA1 | 10572 | 5.45E-09 | -4.51038 |
| PPP1R12B | 4660 | 5.11E-11 | -4.55574 |
| DHRS2 | 10202 | 8.99E-07 | -4.60875 |
| MST1 | 4485 | 6.22E-08 | -5.46682 |
| OSGEPL1 | 64172 | 5.94E-11 | -5.892 |
| KCNJ2 | 3759 | 2.62E-11 | -5.89364 |
| PCLO | 27445 | 8.58E-20 | -7.46315 |
| HDAC4 | 9759 | 1.48E-14 | -8.85811 |
| PURA | 5813 | 4.14E-17 | -9.06875 |
| LPAL2 | 80350 | 6.33E-13 | -10.4039 |
| FGFR3 | 2261 | 5.70E-10 | -11.5079 |
| WNT5A | 7474 | 8.37E-16 | -13.2281 |
| STARD13 | 90627 | 3.70E-22 | -15.7428 |
| FZD4 | 8322 | 3.25E-24 | -15.8328 |
| PHF17 | 79960 | 7.26E-20 | -15.8942 |
| PIK3R1 | 5295 | 1.36E-22 | -21.0785 |
