# Supplementary material for: Regulation of Transcriptional Networks by PKC Isozymes: Identification of c-Rel as a Key Transcription Factor for PKC-Regulated Genes
Source: PLoS One. 2013 Jun 27;8(6):e67319. doi: 10.1371/journal.pone.0067319 (PMC3694964; doi:10.1371/journal.pone.0067319)
Supplement: Table S2 — PKCδ-regulated genes used for PAINT analysis. Differentially expressed genes identified in our previous microarray analysis (15) were filtered as: a) altered in response to PMA by a factor of 2 (−2≤PMA/vehicle≤2); and b) fold-change by PMA is either reduced by ≥ 50% or augmented by ≥ 50% as a consequence of PKCδ RNAi depletion. (DOC) [file pone.0067319.s006.doc]

**Table S2. PKCδ-regulated genes used for PAINT analysis.**

| **Gene Symbol** | **Entrez Gene** | **p-value(PKCdelta * PMA vs. PKCdelta * vehicle)** | **Fold-Change(PKCdelta * PMA vs. PKCdelta * vehicle)** |
| --- | --- | --- | --- |
| DUSP5 | 1847 | 4.37E-24 | 605.843 |
| MAFF | 23764 | 4.24E-31 | 288.541 |
| C8orf4 | 56892 | 1.19E-28 | 223.283 |
| CCL20 | 6364 | 6.44E-41 | 205.778 |
| C3orf52 | 79669 | 6.29E-41 | 140.401 |
| LIPG | 9388 | 6.41E-40 | 138.744 |
| PHLDA1 | 22822 | 2.32E-32 | 114.648 |
| GADD45B | 4616 | 1.75E-33 | 109.887 |
| TNFRSF12A | 51330 | 9.62E-24 | 101.033 |
| MMP12 | 4321 | 5.51E-32 | 100.944 |
| GBP1 | 2633 | 8.27E-27 | 87.9832 |
| KIAA1199 | 57214 | 5.31E-36 | 84.5418 |
| CLDN1 | 9076 | 5.31E-40 | 73.3803 |
| LOC150759 | 150759 | 1.48E-20 | 73.0504 |
| SERPINB2 | 5055 | 2.34E-29 | 69.0852 |
| LBH | 81606 | 6.72E-31 | 66.104 |
| KRT20 | 54474 | 2.88E-19 | 65.6839 |
| C6orf54 | 26236 | 5.75E-44 | 64.4421 |
| EGR3 | 1960 | 5.33E-37 | 61.7552 |
| CACNA1G | 8913 | 6.07E-33 | 61.7359 |
| KHDC1 RP11-257K9.7 | 100129128 80759 | 1.73E-28 | 59.691 |
| FOSL1 | 8061 | 1.92E-40 | 58.8825 |
| IL2RG | 3561 | 1.25E-34 | 58.1727 |
| NAV2 | 89797 | 6.95E-35 | 57.2503 |
| ZEB1 | 6935 | 5.92E-34 | 52.8667 |
| LEPREL1 | 55214 | 2.50E-38 | 52.248 |
| KLF6 | 1316 | 5.04E-23 | 49.7286 |
| ZYX | 7791 | 8.11E-20 | 43.7261 |
| KIAA1462 | 57608 | 2.12E-35 | 42.0577 |
| BCL2A1 | 597 | 6.80E-23 | 41.0672 |
| ALOXE3 | 59344 | 7.59E-32 | 39.8217 |
| BACH2 | 60468 | 4.25E-30 | 31.3885 |
| SFN | 2810 | 5.50E-21 | 31.3409 |
| ALOXE3 | 59344 | 1.19E-35 | 29.7734 |
| DST | 667 | 5.90E-23 | 28.6274 |
| SFN | 2810 | 2.01E-23 | 26.9362 |
| GEM | 2669 | 1.38E-27 | 25.2081 |
| AREG LOC727738 | 374 727738 | 2.59E-18 | 24.0352 |
| DAPK2 | 23604 | 3.16E-25 | 23.7236 |
| LAMB3 | 3914 | 4.49E-28 | 23.6052 |
| ARID3B | 10620 | 1.09E-22 | 22.8919 |
| PLAUR | 5329 | 2.29E-19 | 22.214 |
| CEACAM1 | 634 | 1.10E-28 | 20.6904 |
| NNT | 23530 | 1.59E-26 | 20.322 |
| ANXA2 | 302 | 1.49E-25 | 19.92 |
| ANXA2 | 302 | 6.56E-24 | 18.289 |
| EGLN3 | 112399 | 2.44E-11 | 18.1914 |
| LIF | 3976 | 9.89E-27 | 17.5727 |
| FBXL7 | 23194 | 6.18E-27 | 16.035 |
| DNAH7 | 56171 | 6.11E-18 | 15.4347 |
| IL32 | 9235 | 4.27E-13 | 15.4136 |
| TRAF1 | 7185 | 2.41E-19 | 15.2639 |
| IL1RN | 3557 | 2.90E-12 | 15.2513 |
| AQP3 | 360 | 4.70E-14 | 15.1006 |
| PALLD | 23022 | 1.88E-14 | 14.5063 |
| SYNJ2 | 8871 | 1.56E-23 | 14.4459 |
| MITF | 4286 | 1.68E-19 | 13.9226 |
| ANGPT2 | 285 | 3.22E-12 | 13.4456 |
| NNT | 23530 | 7.75E-15 | 13.0493 |
| PPP1R15A | 23645 | 6.15E-34 | 12.9591 |
| SYNJ2 | 8871 | 3.24E-28 | 12.9467 |
| IL4R | 3566 | 5.01E-22 | 12.5754 |
| CCL7 | 6354 | 1.07E-18 | 12.4481 |
| ANXA2 | 302 | 5.62E-22 | 12.282 |
| TNC | 3371 | 7.22E-13 | 12.2331 |
| KRT34 | 3885 | 5.43E-12 | 12.038 |
| MMP3 | 4314 | 8.43E-15 | 11.8851 |
| GPRC5A | 9052 | 2.08E-20 | 11.7494 |
| ANGPT2 | 285 | 1.97E-08 | 11.6262 |
| CHAC1 | 79094 | 2.62E-19 | 11.4975 |
| LOC388907 LOC647436 RPL5 SNORA66 | 26782 388907 6125 647436 | 5.41E-24 | 11.4692 |
| TCF7 | 6932 | 7.74E-24 | 11.4596 |
| GK | 2710 | 1.32E-11 | 10.9268 |
| RGS4 | 5999 | 4.32E-15 | 10.8745 |
| FBN1 | 2200 | 3.98E-16 | 10.4595 |
| MAST4 | 375449 | 9.24E-18 | 10.3797 |
| SNAI2 | 6591 | 3.59E-23 | 10.3427 |
| MYL9 | 10398 | 2.89E-11 | 10.1906 |
| SMURF1 | 57154 | 6.26E-23 | 9.84286 |
| CAMSAP1 | 157922 | 2.62E-13 | 9.79626 |
| TUBB2A TUBB2B | 347733 7280 | 6.45E-12 | 9.75211 |
| ICOSLG | 23308 | 3.80E-14 | 9.2869 |
| OASL | 8638 | 4.96E-12 | 8.38144 |
| LDLR | 3949 | 1.47E-18 | 8.30123 |
| ITPKC | 80271 | 1.04E-20 | 7.91766 |
| SMURF1 | 57154 | 3.05E-19 | 7.66645 |
| OPTN | 10133 | 4.76E-19 | 7.61669 |
| CLDN3 | 1365 | 1.17E-09 | 7.5923 |
| CLU | 1191 | 7.10E-15 | 7.4398 |
| CRIM1 | 51232 | 7.32E-26 | 7.42601 |
| ETV6 | 2120 | 7.61E-10 | 6.96623 |
| TNIK | 23043 | 9.16E-12 | 6.81149 |
| CCRN4L | 25819 | 5.63E-19 | 6.72496 |
| OBFC2A | 64859 | 1.56E-20 | 6.71156 |
| FAM129A | 116496 | 5.25E-13 | 6.68466 |
| NPAS2 | 4862 | 4.69E-17 | 6.07917 |
| SPAG8 | 26206 | 1.93E-17 | 5.98285 |
| LOC100128443 MAST4 | 100128443 375449 | 2.03E-12 | 5.97764 |
| IL1RL1 | 9173 | 1.15E-09 | 5.71182 |
| ACTA2 | 59 | 9.59E-11 | 5.5507 |
| MAMLD1 | 10046 | 4.45E-12 | 5.50285 |
| PI3 | 5266 | 4.35E-10 | 5.47856 |
| TGFBR3 | 7049 | 3.37E-06 | 5.34288 |
| ETS2 | 2114 | 8.70E-11 | 5.2811 |
| COBLL1 | 22837 | 0.000100785 | 5.25076 |
| TMEM51 | 55092 | 5.28E-16 | 5.20281 |
| PDLIM7 | 9260 | 1.27E-06 | 5.1742 |
| PAEP | 5047 | 7.79E-09 | 5.10543 |
| CD24 | 934 | 3.52E-09 | 5.09272 |
| CLU | 1191 | 1.87E-16 | 5.0836 |
| UPP1 | 7378 | 8.28E-10 | 5.07721 |
| FRMD4B | 23150 | 1.07E-10 | 5.00462 |
| CAMSAP1 | 157922 | 9.42E-10 | 4.92013 |
| OASL | 8638 | 2.92E-11 | 4.91487 |
| ZNF165 | 7718 | 1.03E-19 | 4.72545 |
| PRKCA | 5578 | 2.45E-12 | 4.71905 |
| AGPAT7 | 254531 | 2.08E-11 | 4.64414 |
| SERPINB1 | 1992 | 1.81E-08 | 4.34607 |
| CTH | 1491 | 5.77E-11 | 4.21571 |
| DIXDC1 | 85458 | 3.58E-09 | 4.0584 |
| TAC1 | 6863 | 1.32E-08 | 4.05613 |
| NDRG4 | 65009 | 5.21E-08 | 4.02588 |
| IGSF3 | 3321 | 2.86E-16 | 3.99301 |
| MGLL | 11343 | 2.12E-13 | 3.97429 |
| FEM1B | 10116 | 4.81E-16 | 3.90214 |
| IL1F5 | 26525 | 1.34E-09 | 3.7591 |
| PI3 | 5266 | 2.31E-06 | 3.7118 |
| GPR87 | 53836 | 1.68E-07 | 3.6525 |
| PLAUR | 5329 | 1.27E-05 | 3.59 |
| GCNT1 | 2650 | 3.64E-11 | 3.54296 |
| PTPN12 | 5782 | 1.34E-06 | 3.52034 |
| ROD1 | 9991 | 3.86E-08 | 3.51999 |
| TNFRSF11A | 8792 | 9.76E-10 | 3.51929 |
| RASA2 | 5922 | 1.02E-05 | 3.51023 |
| UGCG | 7357 | 1.25E-17 | 3.47926 |
| PTPN12 | 5782 | 1.40E-07 | 3.47881 |
| SH3GL3 | 6457 | 5.41E-08 | 3.44783 |
| EZR | 7430 | 7.35E-13 | 3.37209 |
| KCNK1 | 3775 | 3.69E-09 | 3.33746 |
| CD4 | 920 | 7.56E-08 | 3.3085 |
| SPHK1 | 8877 | 4.17E-08 | 3.29861 |
| LYST | 1130 | 0.000944393 | 3.26899 |
| RYBP | 23429 | 2.35E-05 | 3.26558 |
| ITPR3 | 3710 | 8.57E-10 | 3.2132 |
| PDZK1 | 5174 | 3.44E-08 | 3.21156 |
| HPCAL1 | 3241 | 8.06E-06 | 3.11736 |
| MAF | 4094 | 1.55E-05 | 3.1046 |
| ELF1 | 1997 | 0.00476107 | 3.04459 |
| NR4A3 | 8013 | 4.50E-05 | 3.0236 |
| ADM | 133 | 1.06E-08 | 2.97782 |
| SOCS2 | 8835 | 2.72E-10 | 2.9579 |
| STX1A | 6804 | 4.34E-08 | 2.785 |
| CSF1 | 1435 | 7.35E-06 | 2.76885 |
| CHRM3 | 1131 | 0.000170693 | 2.70133 |
| FOSB | 2354 | 1.56E-07 | 2.67587 |
| OAS1 | 4938 | 2.50E-08 | 2.63296 |
| GK3P | 2713 | 6.09E-08 | 2.51538 |
| SOX11 | 6664 | 0.000117292 | 2.51125 |
| USP36 | 57602 | 1.83E-06 | 2.49393 |
| C4orf10 | 317648 | 9.40E-05 | 2.41331 |
| TNFAIP2 | 7127 | 0.00149954 | 2.35697 |
| F11R | 50848 | 8.26E-09 | 2.34404 |
| ARHGAP22 | 58504 | 0.00124981 | 2.31186 |
| PLAGL2 | 5326 | 2.91E-06 | 2.31105 |
| FGF2 | 2247 | 0.000300926 | 2.31008 |
| MARCKS | 4082 | 0.0326236 | 2.2572 |
| KRT81 | 3887 | 5.51E-07 | 2.20327 |
| MCPH1 | 79648 | 0.00178412 | 2.17172 |
| MRC1 /// MRC1L1 | 414308 /// 4360 | 0.000319971 | 2.09239 |
| ARHGAP25 | 9938 | 0.00676623 | 2.08242 |
| GK | 2710 | 0.000296181 | 2.06999 |
| BTRC | 8945 | 5.19E-08 | 2.06836 |
| C15orf39 | 56905 | 0.0022235 | 2.06623 |
| CLCF1 | 23529 | 0.00518718 | 2.05484 |
| C14orf139 | 79686 | 3.25E-06 | 2.05364 |
| KRT15 | 3866 | 0.000847767 | 2.01273 |
| IL1RN | 3557 | 0.00275587 | 2.0024 |
| DAPK3 | 1613 | 0.042403 | 1.98137 |
| GK | 2710 | 0.00108816 | 1.98108 |
| GPR3 | 2827 | 0.00211446 | 1.95535 |
| ELL2 | 22936 | 0.00103274 | 1.94862 |
| CDKN2A | 1029 | 5.59E-11 | 1.94775 |
| VPS37B | 79720 | 0.000334784 | 1.92179 |
| LUZP1 | 7798 | 0.000274351 | 1.9123 |
| STK10 | 6793 | 0.000692771 | 1.90758 |
| GAL | 51083 | 0.000722885 | 1.85303 |
| SVIL | 6840 | 0.00251049 | 1.84983 |
| EGR1 | 1958 | 0.0141036 | 1.82828 |
| BTRC | 8945 | 0.000143018 | 1.82609 |
| OXTR | 5021 | 5.52E-06 | 1.82508 |
| PRKCA | 5578 | 0.00119665 | 1.82246 |
| TMOD1 | 7111 | 0.000824303 | 1.81825 |
| RASSF2 | 9770 | 0.0351475 | 1.80677 |
| TTC9 | 23508 | 0.00138487 | 1.78045 |
| SPSB1 | 80176 | 0.000150492 | 1.77996 |
| RHOF | 54509 | 0.000542593 | 1.75741 |
| CD55 | 1604 | 0.00370907 | 1.72674 |
| C6orf155 | 79940 | 0.000221853 | 1.71353 |
| GNA13 | 10672 | 0.00864301 | 1.71191 |
| ARHGEF2 | 9181 | 0.000636818 | 1.66832 |
| SOX11 | 6664 | 0.000770428 | 1.65867 |
| NOV | 4856 | 0.0271863 | 1.61739 |
| SLC22A1 | 6580 | 0.00171603 | 1.61635 |
| GPC1 | 2817 | 0.00215564 | 1.61633 |
| SMAD3 | 4088 | 0.148974 | 1.58513 |
| SPRY2 | 10253 | 0.0106583 | 1.57343 |
| DOCK10 | 55619 | 0.00213735 | 1.56886 |
| KIAA0999 | 23387 | 0.00246921 | 1.56697 |
| FERMT2 | 10979 | 0.0840471 | 1.53591 |
| CAV1 | 857 | 0.00570478 | 1.53492 |
| PPP1R15A | 23645 | 0.0174086 | 1.51627 |
| APOBEC3A | 200315 | 0.00530555 | 1.48176 |
| AGPAT7 | 254531 | 0.00523139 | 1.43717 |
| RNF19A | 25897 | 0.153045 | 1.42743 |
| BCL2L14 | 79370 | 0.0687995 | 1.40186 |
| CSNK1G1 | 53944 | 0.0437813 | 1.39294 |
| KIAA1466 | 57612 | 0.116577 | 1.39243 |
| ARHGAP25 | 9938 | 0.11748 | 1.37086 |
| CTSB | 1508 | 0.044777 | 1.36195 |
| MAP2 | 4133 | 0.00869584 | 1.35376 |
| GK /// GK3P | 2710 /// 2713 | 0.103173 | 1.34786 |
| TRPC1 | 7220 | 0.0699873 | 1.32433 |
| SOCS1 | 8651 | 0.0104558 | 1.31112 |
| DENND3 | 22898 | 0.0977987 | 1.30471 |
| CDKN2A | 1029 | 0.0377028 | 1.30459 |
| SLC15A1 | 6564 | 0.0330441 | 1.2857 |
| SOX11 | 6664 | 0.256764 | 1.27558 |
| TPBG | 7162 | 0.0759577 | 1.26595 |
| TRPC1 | 7220 | 0.265529 | 1.24457 |
| SFN | 2810 | 0.421996 | 1.2425 |
| PDGFA | 5154 | 0.363697 | 1.24012 |
| FLJ42627 | 645644 | 0.338662 | 1.23751 |
| IL1B | 3553 | 0.296595 | 1.23625 |
| MAP3K1 | 4214 | 0.201656 | 1.23019 |
| FAM46A | 55603 | 0.229043 | 1.22647 |
| SEMA4C | 54910 | 0.0180291 | 1.22344 |
| MYO9B | 4650 | 0.366527 | 1.20856 |
| CD24 | 934 | 0.193765 | 1.20698 |
| NPR2 | 4882 | 0.279815 | 1.20409 |
| LTBP1 | 4052 | 0.266925 | 1.19568 |
| C6orf124 /// LOC729439 | 653483 /// 729439 | 0.147845 | 1.19444 |
| LAMC2 | 3918 | 0.43569 | 1.16899 |
| DTNA | 1837 | 0.341315 | 1.14796 |
| ABCA4 | 24 | 0.446763 | 1.14566 |
| C4orf6 | 10141 | 0.425785 | 1.14492 |
| EMP1 | 2012 | 0.47207 | 1.13412 |
| MET | 4233 | 0.447862 | 1.13399 |
| SLC1A3 | 6507 | 0.351575 | 1.13266 |
| IL1B | 3553 | 0.563509 | 1.13226 |
| GP1BB SEPT5 | 2812 5413 | 0.638445 | 1.12875 |
| KIAA1622 | 57718 | 0.532657 | 1.11924 |
| FBN1 | 2200 | 0.503747 | 1.11555 |
| C13orf18 LOC728970 | 728970 80183 | 0.528043 | 1.10578 |
| CCL22 | 6367 | 0.712619 | 1.10352 |
| BCL2 | 596 | 0.42217 | 1.1031 |
| RASSF9 | 9182 | 0.606821 | 1.09786 |
| SH3TC1 | 54436 | 0.481947 | 1.09054 |
| TLE1 | 7088 | 0.480019 | 1.08771 |
| VRK3 | 51231 | 0.581301 | 1.07143 |
| CCDC21 | 64793 | 0.638994 | 1.06839 |
| C6orf54 | 26236 | 0.605549 | 1.06749 |
| SELPLG | 6404 | 0.646219 | 1.06614 |
| PLAU | 5328 | 0.780209 | 1.06095 |
| CEACAM1 | 634 | 0.751383 | 1.05739 |
| SPRR1A | 6698 | 0.811993 | 1.05207 |
| GDPD5 | 81544 | 0.712684 | 1.04607 |
| IL1RN | 3557 | 0.907218 | 1.0391 |
| ZEB1 | 6935 | 0.631992 | 1.03877 |
| CEACAM1 | 634 | 0.872745 | 1.02786 |
| KRT75 | 9119 | 0.941329 | 1.02602 |
| TNFRSF11B | 4982 | 0.893684 | 1.02276 |
| RAP2A RAP2B | 5911 5912 | 0.928428 | 1.01894 |
| LOC339047 LOC348162 LOC642778 LOC729602 | 339047 348162 642778 729602 | 0.957656 | 1.01829 |
| AKR1B10 | 57016 | 0.774012 | 1.01609 |
| MRPS11 | 64963 | 0.913179 | 1.01607 |
| INDO | 3620 | 0.91222 | 1.01579 |
| IL1F9 | 56300 | 0.961538 | 1.01424 |
| BCL2L11 | 10018 | 0.807637 | -1.02572 |
| REEP1 | 65055 | 0.611552 | -1.05956 |
| PCDH11Y | 83259 | 0.864632 | -1.06692 |
| KIAA0922 | 23240 | 0.62683 | -1.0734 |
| ZNF294 | 26046 | 0.347746 | -1.08804 |
| RICS | 9743 | 0.558856 | -1.10022 |
| ERCC6L | 54821 | 0.456977 | -1.13322 |
| CAMTA1 | 23261 | 0.351739 | -1.16186 |
| RICS | 9743 | 0.0389146 | -1.16897 |
| SLC17A5 | 26503 | 0.810072 | -1.18951 |
| SOCS5 | 9655 | 0.177745 | -1.3463 |
| CHST10 | 9486 | 0.0086843 | -1.55487 |
| KIF5C | 3800 | 0.220991 | -1.70706 |
| ADAM7 | 8756 | 0.0411186 | -1.74444 |
| FZD1 | 8321 | 6.61E-08 | -1.75885 |
| EPOR | 2057 | 5.90E-06 | -1.78622 |
| SETBP1 | 26040 | 4.30E-05 | -1.88683 |
| GUSBP1 | 153561 | 0.0163385 | -2.03554 |
| HJURP | 55355 | 1.70E-09 | -2.16685 |
| RPGRIP1L | 23322 | 2.07E-05 | -2.16746 |
| SLC26A2 | 1836 | 0.0656833 | -2.18154 |
| MDM1 | 56890 | 7.36E-06 | -2.28481 |
| MYB | 4602 | 6.66E-06 | -2.28509 |
| E2F8 | 79733 | 2.04E-05 | -2.30519 |
| NNMT | 4837 | 1.93E-06 | -2.44168 |
| CXorf34 | 79979 | 0.323764 | -2.44974 |
| PDE9A | 5152 | 0.000450536 | -2.5983 |
| SOX12 | 6666 | 1.26E-13 | -2.67016 |
| AURKA | 6790 | 2.58E-18 | -2.77597 |
| LOC652637 WHDC1L1 | 339005 652637 | 8.23E-10 | -2.90843 |
| PALMD | 54873 | 2.57E-07 | -2.96025 |
| AURKA | 6790 | 3.85E-18 | -2.98382 |
| TTBK2 | 146057 | 4.19E-07 | -3.13688 |
| ASPM | 259266 | 1.21E-13 | -3.1539 |
| EPOR | 2057 | 6.45E-09 | -3.23777 |
| ADRA2A | 150 | 4.69E-09 | -3.24259 |
| ZBTB16 | 7704 | 3.17E-09 | -3.41609 |
| ENOX2 | 10495 | 7.50E-11 | -3.61028 |
| CDK5RAP2 | 55755 | 2.96E-07 | -3.61535 |
| GGA2 | 23062 | 3.17E-12 | -3.64819 |
| PPP1R3D | 5509 | 1.74E-15 | -4.1307 |
| TMEM140 | 55281 | 7.43E-10 | -4.35472 |
| KIF2C | 11004 | 1.90E-07 | -4.45154 |
| OSR2 | 116039 | 5.59E-13 | -4.636 |
| H2AFJ | 55766 | 1.58E-11 | -4.72323 |
| LGR4 | 55366 | 1.75E-10 | -4.99919 |
| PIK3R3 | 8503 | 8.97E-14 | -5.71321 |
| GRHL2 | 79977 | 1.71E-24 | -5.73562 |
| B3GNT1 | 11041 | 2.55E-14 | -6.46598 |
| KIF20A | 10112 | 9.51E-13 | -6.57226 |
| TRIM48 | 79097 | 1.39E-08 | -6.81481 |
| SLITRK3 | 22865 | 8.33E-15 | -6.96534 |
| SEMA6A | 57556 | 6.86E-11 | -8.02895 |
| SMAD6 | 4091 | 1.27E-17 | -8.34856 |
| C8orf51 | 78998 | 9.44E-19 | -8.48837 |
| HOXC4 HOXC6 | 3221 3223 | 4.15E-27 | -8.74376 |
| STARD13 | 90627 | 7.21E-20 | -10.7944 |
| SESN1 | 27244 | 2.49E-20 | -11.8623 |
| FGFR3 | 2261 | 2.48E-10 | -12.476 |
| PSRC1 | 84722 | 5.57E-20 | -13.7051 |
| LIN7B | 64130 | 1.54E-17 | -15.0948 |
| C5orf4 | 10826 | 1.91E-22 | -15.1944 |
| FZD4 | 8322 | 2.02E-24 | -16.4039 |
| ADRB2 | 154 | 3.25E-24 | -16.6854 |
| SPRY1 | 10252 | 3.47E-20 | -17.4948 |
