# Supplementary material for: Regulation of Transcriptional Networks by PKC Isozymes: Identification of c-Rel as a Key Transcription Factor for PKC-Regulated Genes
Source: PLoS One. 2013 Jun 27;8(6):e67319. doi: 10.1371/journal.pone.0067319 (PMC3694964; doi:10.1371/journal.pone.0067319)
Supplement: Table S3 — PKCε-regulated genes used for PAINT analysis. Differentially expressed genes identified in our previous microarray analysis (15) were filtered as: a) altered in response to PMA by a factor of 2 (−2≤PMA/vehicle≤2); and b) fold-change by PMA is either reduced by ≥ 50% or augmented by ≥ 50% as a consequence of PKCε RNAi depletion. (DOC) [file pone.0067319.s007.doc]

**Table S3. PKCε-regulated genes used for PAINT analysis.**

| **Gene Symbol** | **Entrez Gene** | **p-value(PKCepsilon * PMA vs. PKCepsilon * vehicle)** | **Fold-Change(PKCepsilon * PMA vs. PKCepsilon * vehicle)** |
| --- | --- | --- | --- |
| ARL14 | 80117 | 1.18E-37 | 164.088 |
| CXCL11 | 6373 | 8.15E-41 | 150.049 |
| LOC150759 | 150759 | 6.27E-21 | 81.1171 |
| CYR61 | 3491 | 9.02E-25 | 78.7102 |
| BIRC3 | 330 | 1.05E-26 | 75.6516 |
| PI3 | 5266 | 5.53E-22 | 56.5498 |
| MMP3 | 4314 | 6.82E-22 | 54.3344 |
| PI3 | 5266 | 1.89E-19 | 50.8022 |
| SFN | 2810 | 7.09E-23 | 48.6443 |
| NDRG4 | 65009 | 2.75E-21 | 48.1821 |
| RGS4 | 5999 | 2.57E-22 | 48.1257 |
| EGR1 | 1958 | 3.57E-26 | 41.1426 |
| MAP3K8 | 1326 | 1.75E-26 | 36.8884 |
| ANKRD1 | 27063 | 3.32E-19 | 35.9988 |
| EBI2 | 1880 | 3.91E-19 | 33.9365 |
| CLCF1 | 23529 | 2.35E-17 | 32.8541 |
| ANXA2 | 302 | 4.31E-28 | 32.8309 |
| NR4A3 | 8013 | 3.65E-17 | 30.5885 |
| KRT81 | 3887 | 1.70E-26 | 30.3645 |
| FBXL7 | 23194 | 1.22E-29 | 26.2069 |
| KLF5 | 688 | 6.06E-25 | 24.4881 |
| FABP5 FABP5L2 FABP5L7 | 2171 728641 729163 | 4.22E-22 | 23.7628 |
| CSF1 | 1435 | 8.60E-19 | 22.7506 |
| ANXA2 | 302 | 1.92E-25 | 22.5072 |
| KRT20 | 54474 | 6.53E-15 | 22.1502 |
| EGLN3 | 112399 | 4.96E-12 | 21.584 |
| MITF | 4286 | 9.47E-22 | 21.022 |
| FOSB | 2354 | 4.74E-22 | 20.6724 |
| FOS | 2353 | 4.24E-16 | 17.9569 |
| OASL | 8638 | 7.17E-16 | 17.139 |
| MBD2 | 8932 | 9.45E-21 | 15.7288 |
| F2RL1 | 2150 | 4.00E-18 | 14.0743 |
| SCHIP1 | 29970 | 1.63E-18 | 13.7828 |
| GK | 2710 | 1.39E-12 | 13.3133 |
| TNC | 3371 | 8.22E-13 | 12.0987 |
| CAV1 | 857 | 6.73E-20 | 12.0909 |
| COBLL1 | 22837 | 7.60E-13 | 11.611 |
| C13orf15 | 28984 | 1.00E-13 | 10.9478 |
| EGR1 | 1958 | 1.40E-12 | 10.77 |
| RASSF8 | 11228 | 4.52E-13 | 10.6198 |
| NOV | 4856 | 7.17E-14 | 10.4117 |
| KRT15 | 3866 | 1.57E-14 | 9.73283 |
| NR4A2 | 4929 | 1.40E-13 | 9.21831 |
| RGS2 | 5997 | 1.04E-14 | 9.03592 |
| TUBB2A TUBB2B | 347733 7280 | 1.92E-11 | 8.9344 |
| CHAC1 | 79094 | 1.12E-17 | 8.93185 |
| PLAU | 5328 | 7.49E-13 | 8.80818 |
| RASGRP1 | 10125 | 1.71E-16 | 8.77299 |
| IL1F9 | 56300 | 4.59E-09 | 8.74191 |
| SERPINB1 | 1992 | 9.51E-13 | 8.56443 |
| GAL | 51083 | 2.22E-15 | 8.18625 |
| SNAI2 | 6591 | 2.08E-21 | 8.08122 |
| PDLIM5 | 10611 | 3.87E-12 | 7.85231 |
| IL1B | 3553 | 1.05E-11 | 7.45619 |
| ANGPT2 | 285 | 3.92E-09 | 7.25581 |
| OASL | 8638 | 5.19E-14 | 7.24783 |
| RELB | 5971 | 3.71E-17 | 7.20583 |
| PLEKHO1 | 51177 | 8.35E-12 | 7.14509 |
| NR4A2 | 4929 | 1.28E-15 | 7.13491 |
| BCL2L14 | 79370 | 2.29E-13 | 6.97302 |
| IL6ST | 3572 | 1.37E-08 | 6.56948 |
| CD83 | 9308 | 7.55E-09 | 6.29118 |
| CAMSAP1 | 157922 | 2.64E-10 | 5.34062 |
| BLZF1 | 8548 | 2.35E-13 | 5.32244 |
| FJX1 | 24147 | 3.53E-12 | 5.26637 |
| PDLIM5 | 10611 | 1.83E-09 | 5.04636 |
| SOD2 | 6648 | 2.13E-06 | 5.00004 |
| TNFRSF11B | 4982 | 4.78E-08 | 4.81745 |
| KRT75 | 9119 | 8.74E-05 | 4.54406 |
| RASSF9 | 9182 | 6.76E-10 | 4.25873 |
| C4orf10 | 317648 | 1.25E-08 | 4.24785 |
| SFN | 2810 | 3.27E-06 | 4.24448 |
| SH3GL3 | 6457 | 1.94E-09 | 4.18383 |
| EIF2AK3 | 9451 | 8.24E-16 | 4.11218 |
| SLC9A2 | 6549 | 0.005674 | 3.73405 |
| STK10 | 6793 | 3.25E-08 | 3.31773 |
| ARHGAP22 | 58504 | 2.15E-05 | 3.19382 |
| SOX11 | 6664 | 3.55E-06 | 3.18852 |
| MYO1B | 4430 | 2.37E-08 | 3.17944 |
| RGS5 | 8490 | 1.30E-06 | 3.13314 |
| GK | 2710 | 8.22E-07 | 3.10048 |
| SOX11 | 6664 | 2.15E-09 | 2.90823 |
| MARCKS | 4082 | 0.006325 | 2.88637 |
| GK | 2710 | 1.79E-06 | 2.78882 |
| GK3P | 2713 | 1.69E-08 | 2.65877 |
| SLC7A1 | 6541 | 0.014532 | 2.64991 |
| NPR2 | 4882 | 1.07E-06 | 2.64978 |
| CCL22 | 6367 | 0.001219 | 2.52082 |
| C14orf139 | 79686 | 2.37E-08 | 2.51589 |
| ARHGEF2 | 9181 | 9.57E-08 | 2.45166 |
| SMAD3 | 4088 | 0.007666 | 2.4088 |
| TNFAIP2 | 7127 | 0.001461 | 2.36237 |
| RP4-724E16.2 | 80089 | 0.002946 | 2.30224 |
| CEACAM1 | 634 | 1.96E-05 | 2.28228 |
| MET | 4233 | 1.54E-05 | 2.24076 |
| BTRC | 8945 | 2.84E-06 | 2.18144 |
| SVIL | 6840 | 0.000212 | 2.17543 |
| GNA13 | 10672 | 0.00037 | 2.13233 |
| SOX11 | 6664 | 0.000985 | 2.12195 |
| PRKCA | 5578 | 0.000163 | 2.04622 |
| LUZP1 | 7798 | 0.000253 | 1.92067 |
| FERMT2 | 10979 | 0.015426 | 1.84577 |
| GPR3 | 2827 | 0.008243 | 1.76328 |
| ELL2 | 22936 | 0.288511 | 1.70099 |
| CDH1 | 999 | 0.086717 | 1.65989 |
| DAPK3 | 1613 | 0.133777 | 1.64733 |
| CHRM3 | 1131 | 0.048453 | 1.62884 |
| ELL2 | 22936 | 0.0144 | 1.61964 |
| LTBP1 | 4052 | 0.009073 | 1.54549 |
| CD24 | 934 | 0.015991 | 1.43057 |
| ATP6V0A1 | 535 | 0.229137 | 1.31972 |
| RANGAP1 | 5905 | 0.206162 | 1.29332 |
| LOC339047 LOC348162 LOC642778 LOC729602 | 339047 348162 642778 729602 | 0.744522 | 1.11776 |
| RNF24 | 11237 | 0.619051 | 1.11001 |
| ZEB1 | 6935 | 0.217308 | 1.10383 |
| AMIGO2 | 347902 | 0.598285 | 1.09313 |
| SLC17A5 | 26503 | 0.907342 | 1.08765 |
| DTNA | 1837 | 0.67315 | 1.06278 |
| SELPLG | 6404 | 0.674962 | 1.06024 |
| S100A4 | 6275 | 0.491739 | -1.11245 |
| PCDH11Y | 83259 | 0.719729 | -1.14617 |
| CXorf34 | 79979 | 0.864342 | -1.16673 |
| SRGAP3 | 9901 | 0.27789 | -1.25539 |
| RAB6B | 51560 | 0.431388 | -1.27594 |
| MBD5 | 55777 | 0.000334 | -1.64475 |
| C5orf4 | 10826 | 1.32E-05 | -2.58972 |
| SOX12 | 6666 | 1.99E-14 | -2.83737 |
| PDE9A | 5152 | 0.000136 | -2.86856 |
| BRD3 | 8019 | 3.15E-07 | -3.2723 |
| ZBTB48 | 3104 | 2.75E-08 | -3.33803 |
| GIT2 | 9815 | 4.83E-11 | -3.4424 |
| TUT1 | 64852 | 8.00E-07 | -3.48211 |
| F5 | 2153 | 1.57E-07 | -3.4847 |
| PRPF4 | 9128 | 1.97E-08 | -3.51531 |
| RNF146 | 81847 | 5.41E-10 | -3.51573 |
| RNF113A | 7737 | 1.47E-18 | -3.77266 |
| ZNF589 | 51385 | 5.25E-13 | -3.83062 |
| IPO8 | 10526 | 2.43E-06 | -3.96958 |
| ADAM7 | 8756 | 1.15E-07 | -4.24495 |
| ZNF235 | 9310 | 4.93E-10 | -4.52816 |
| TMEM140 | 55281 | 3.03E-10 | -4.5918 |
| DHRS2 | 10202 | 8.98E-07 | -4.60902 |
| LIPT1 | 51601 | 3.25E-19 | -4.88 |
| LRRC36 | 55282 | 3.41E-10 | -5.00901 |
| ZNF74 | 7625 | 8.10E-11 | -5.1068 |
| ZNF227 | 7770 | 3.68E-13 | -5.39549 |
| MAST3 | 23031 | 3.87E-15 | -5.43295 |
| ZNF211 | 10520 | 6.71E-20 | -5.64088 |
| KLHL20 | 27252 | 1.97E-16 | -5.70408 |
| ZNF177 | 7730 | 1.39E-17 | -5.8189 |
| CSTF1 | 1477 | 5.74E-14 | -5.85766 |
| GPER | 2852 | 1.29E-13 | -6.0348 |
| C2orf44 | 80304 | 2.62E-17 | -6.19509 |
| PQBP1 | 10084 | 4.15E-10 | -6.51454 |
| E4F1 | 1877 | 5.38E-11 | -6.61255 |
| CYorf15B | 84663 | 5.77E-17 | -6.79898 |
| ZMYND8 | 23613 | 9.23E-16 | -6.86493 |
| LGR4 | 55366 | 5.51E-13 | -7.23958 |
| MR1 | 3140 | 9.09E-21 | -7.25804 |
| FRY | 10129 | 2.18E-09 | -7.8886 |
| B3GNT1 | 11041 | 5.33E-16 | -8.23165 |
| MTRF1 | 9617 | 1.18E-19 | -8.30465 |
| ID1 | 3397 | 1.08E-13 | -8.43744 |
| HOXC4 HOXC6 | 3221 3223 | 5.83E-27 | -8.57641 |
| KIF20A | 10112 | 1.59E-14 | -8.63215 |
| ADRA2A | 150 | 1.28E-16 | -8.6434 |
| KIAA0485 | 57235 | 3.04E-08 | -9.06904 |
| C5orf4 | 10826 | 7.72E-22 | -13.7144 |
| SEMA6A | 57556 | 9.01E-14 | -13.8298 |
| STARD13 | 90627 | 1.62E-22 | -16.77 |
| FZD4 | 8322 | 3.44E-25 | -18.7944 |
| LIN7B | 64130 | 1.07E-18 | -18.817 |
| ADRB2 | 154 | 1.35E-27 | -31.8889 |
| TXNIP | 10628 | 2.26E-26 | -52.0495 |
